# Enhancement of TKI sensitivity in lung adenocarcinoma through m6A-dependent translational repression of Wnt signaling by circ-FBXW7

**DOI:** 10.1186/s12943-023-01811-0

**Published:** 2023-07-01

**Authors:** Kai Li, Zi-Yang Peng, Rui Wang, Xiang Li, Ning Du, Da-Peng Liu, Jia Zhang, Yun-Feng Zhang, Lei Ma, Ye Sun, Shou-Ching Tang, Hong Ren, Yi-Ping Yang, Xin Sun

**Affiliations:** 1 Department of Otorhinolaryngology-Head and Neck Surgery, the First Affiliated Hospital of Xi’an Jiaotong University, Xi’an, 710061 Shaanxi Province China; 2 School of Future Technology, National Local Joint Engineering Research Center for Precision Surgery & Regenerative Medicine, Xi’an Jiaotong University, Xi’an City, 710061 Shaanxi Province China; 3 Department of Thoracic Surgery, Department of Thoracic Surgery & Oncology, the First Affiliated Hospital of Xi’an Jiaotong University, Cancer Centre, Xi’an City, 710061 Shaanxi Province China; 4 Cancer Biology Program, University of Hawai’i Cancer Center, 701 Ilalo Street, Honolulu, HI 96813 USA; 5 Department of Anesthesiology & Perioperative Medicine, Operating Centre, the First Affiliated Hospital of Xi’an Jiaotong University, Xi’an City, 710061 Shaanxi Province China; 6 Department of Anesthesiology & Operation, Operating Centre, the First Affiliated Hospital of Xi’an Jiaotong University, Xi’an City, 710061 Shaanxi Province China; 7 LSU School of Medicine, LSU-LCMC Cancer Center, New Orleans, Louisiana 70112 USA; 8 Department of Radiotherapy, Shaanxi Provincial Tumor Hospital, Shaanxi 710061 Xi’an City, China; 9 Department of Pathology, Anatomy & Cell Biology, Sidney Kimmel Cancer Center, Thomas Jefferson University, Philadelphia, PA 19107 USA

**Keywords:** Translated circRNAs, m6A modification, Tyrosine kinase inhibitor, Lung adenocarcinoma, Therapy resistance

## Abstract

**Background:**

Tyrosine kinase inhibitors (TKIs) that specifically target mutational points in the EGFR gene have significantly reduced suffering and provided greater relief to patients with lung adenocarcinoma (LUAD). The third-generation EGFR-TKI, Osimertinib, has been successfully employed in clinical treatments to overcome resistance to both original and acquired T790M and L858R mutational points. Nevertheless, the issue of treatment failure response has emerged as an insurmountable problem.

**Methods:**

By employing a combination of multiple and integrated approaches, we successfully identified a distinct population within the tumor group that plays a significant role in carcinogenesis, resistance, and recurrence. Our research suggests that addressing TKI resistance may involve targeting the renewal and repopulation of stem-like cells. To investigate the underlying mechanisms, we conducted RNA Microarray and m6A Epi-Transcriptomic Microarray analyses, followed by assessment of transcription factors. Additionally, we specifically designed a tag to detect the polypeptide circRNA-AA, and its expression was confirmed through m6A regulations.

**Results:**

We initially identified unique molecular signatures present in cancer stem cells that contributed to poor therapeutic responses. Activation of the alternative Wnt pathway was found to sustain the renewal and resistant status of these cells. Through bioinformatics analysis and array studies, we observed a significant decrease in the expression of circFBXW7 in Osimertinib-resistant cell lines. Notably, the abnormal expression pattern of circFBXW7 determined the cellular response to Osimertinib. Functional investigations revealed that circFBXW7 inhibits the renewal of cancer stem cells and resensitizes both resistant LUAD cells and stem cells to Osimertinib. In terms of the underlying mechanism, we discovered that circFBXW7 can be translated into short polypeptides known as circFBXW7-185AA. These polypeptides interact with β-catenin in an m6A-dependent manner. This interaction leads to reduced stability of β-catenin by inducing subsequent ubiquitination, thereby suppressing the activation of canonical Wnt signaling. Additionally, we predicted that the m6A reader, YTHDF3, shares common binding sites with hsa-Let-7d-5p. Enforced expression of Let-7d post-transcriptionally decreases the levels of YTHDF3. The repression of Let-7d by Wnt signaling releases the stimulation of m6A modification by YTHDF3, promoting the translation of circFBXW7-185AA. This creates a positive feedback loop contributing to the cascade of cancer initiation and promotion.

**Conclusions:**

Our bench study, in vivo experiments, and clinical validation have unequivocally shown that circFBXW7 effectively inhibits the abilities of LUAD stem cells and reverses resistance to TKIs by modulating Wnt pathway functions through the action of circFBXW7-185AA on β-catenin ubiquitination and inhibition. The regulatory role of circRNA in Osimertinib treatment has been rarely reported, and our findings reveal that this process operates under the influence of m6A modification. These results highlight the tremendous potential of this approach in enhancing therapeutic strategies and overcoming resistance to multiple TKI treatments.

**Supplementary Information:**

The online version contains supplementary material available at 10.1186/s12943-023-01811-0.

## Introduction

Therapy response plays a critical role in assessing treatment efficacy and survival outcomes. However, it is also essential to consider the impact on quality of life when evaluating specific treatment strategies. The field of lung cancer treatment has undergone significant advancements, thanks to the identification of novel targets and continuous refinement of therapeutic approaches. Particularly noteworthy is the emergence of targeted therapies, such as tyrosine kinase inhibitors (TKIs), which have gained recognition for their ability to elicit improved responses with fewer side effects [1–3]. The development of third-generation TKIs has propelled them to the forefront of first-line therapy for patients with sensitive EGFR mutations, surpassing the efficacy of earlier generations [4, 5]. Despite the significant advantages demonstrated by third-generation TKI Osimertinib in terms of prolonged survival and reduced recurrence risk, patients undergoing long-term treatment still encounter resistance and disease progression. Finding ways to enhance therapeutic effects and overcome resistance in non-responsive cells holds great promise for improving outcomes and providing patients with a life of freedom, joy, and extended survival.

Stem cells serve as the foundation of cancer, capable of perpetuating the dissemination of cancer cells throughout the tumor microenvironment, while maintaining their own steady and immortal existence [6–8]. Despite years of debate, the distinctive and robust signatures of stem cells represent a crucial and promising area of study. The manner in which cells divide plays a pivotal role in determining the expansion of cancer cells [9–11]. Symmetric division involving differentiation signatures leads to the depletion of stem cells. Conversely, the symmetric division involving self-renewal and the asymmetric division contribute to the expansion of the stem cell pool, resulting in rapid tumor progression and therapy resistance. Oncogenic signaling pathways, such as Wnt signaling, consistently participate in critical malignant processes, with key factors within the Wnt pathway indicating poorer survival outcomes [12, 13]. Additionally, we have identified the significant potential of the Wnt pathway in generating the stem cell population and driving therapy resistance [14–16]. By constraining the division process and weakening the defense system dominated by stem cells, it becomes possible to target and eradicate the entire cancer population. The biased expression and activation of Wnt signaling in daughter stem cells determine cellular fate and the resurgence of the tumor population, making tailored modulation of Wnt signaling an essential strategy for repressing stem cell activity.

CircRNAs are a subclass of non-coding RNAs known for their unique circular structures, which endow them with diverse and continuous functional roles across various biological processes [17–19]. While the involvement of circRNAs in cancer initiation, progression, resistance, and recurrence has been extensively studied, their specific roles in lung cancer treatment, particularly their interactions with TKIs, remain poorly understood. In an attempt to uncover the potential functions of specific circRNAs in mediating resistance or sensitivity, we embarked on an investigation to determine the consequences of manipulating circRNA expression and functions in lung cancer stem cell populations. Epigenetic regulations, including M6A modifications and other specific manipulations, serve as crucial switches that initiate or terminate the functions of various RNAs, offering fascinating insights into the intricate world of non-coding RNAs. Recently, we have made significant strides in understanding the upstream signaling pathways governing M6A modifications in non-coding RNA generation. Through this study, we aim to shed new light on the hidden realm of non-coding RNAs and instill hope in patients suffering from advanced lung cancer.

## Materials and methods

### The flow-cytometry and FACSAria analysis and ALDH1 based flow seperation

Cancer cells were processed to obtain a single-cell suspension through enzymatic digestion and followed by mechanical dissociation. Add the fluorescently labeled antibodies to the single-cell suspension and incubate the mixture for a specified time at an appropriate temperature (CD133 Monoclonal Antibody (TMP4), PE, eBioscience™, CD133 CD44 Monoclonal Antibody (IM7), FITC, eBioscience™). After incubation, wash the cells to remove any unbound antibodies or debris by centrifugation and resuspending the cells in a buffer solution. Proper washing helps to reduce background noise and ensure accurate analysis, and then calibrate the flow cytometry machine using appropriate calibration beads or particles in FLOW FACILITY ROOM.

A gating strategy is applied to exclude debris, cell aggregates, and non-viable cells. In machine linked computer, we firstly draw the gates to indicate the regions in scatter plots or histogram plots that define the boundaries of specific cell subsets, by using appropriate control samples separately. The Flow Cytometry Compensation Beads (Comp-Bead 3 Population (5.5 µm) Kit, NBP3-00497, Novus Biologicals) was applied to set voltages and gating parameters for obtaining accurate fluorescence signal. The beads serve as a quality control for fluorochrome-conjugated monoclonal antibodies minimizing the effects of spectral spillover between multiple fluorochromes and flow cytometer channels. Data on fluorescence intensity and other parameters are collected for each cell.

### The ALDH1A1 marker based flow analyzing and separating

For ALDH1 based flow analysis, grouped cells are collected and prepared into a single-cell suspension, and then are incubated with fluorescently labeled antibodies that specifically recognize ALDH1 (ALDEFLUOR™ Assay Kit, STEMCELL™). These antibodies bind to ALDH1 molecules present on the cell surface or intracellularly, and the analysis was done in FLOW FACILITY ROOM. The fluorescence intensity is recorded for each cell, allowing for the identification and quantification of ALDH1-positive cells. The resulting data can be analyzed using specialized software to determine the proportion of ALDH1-positive cells within the population and to compare their characteristics with ALDH1-negative cells. This method was also introduce in detail at https://www.stemcell.com/basic-facs-about-aldefluor-a-guide-to-successful-flow-cytometry-analysis.html.

### Cancer cells divisions and staining assay

Cell division is a fundamental process by which cells reproduce and create new cells, however, in cancer cells, these regulatory mechanisms become disrupted, leading to uncontrolled cell division and tumor formation [20–23]. Cancer cells can undergo two types of cell divisions: symmetric and asymmetric division. As to the EdU labeling assay, cells of different groups were seeded into chamber slides with RS glass (Nunc, Thermo Scientific) after treatments. EdU intensity was detected after cells being incubated with 10uM EdU for 24 h. Detection was performed with using Click-iT Plus EdU Alexa imaging Kit (MP 10637, Life Technologies) [14, 16, 24–26]. The fluorescence images were obtained using an Olympus microscope.

### M6A methylation quantification assay

The m6A methylation status of cells detected using the m6A RNA Methylation Quantification Kit (Epigentek, Cat#P-9005–113) according to the manufacturer’s instructions. In brief, 200 ng of total RNA was used as an input respectively. Then RNA samples were captured and detected by spectrophotometer (Bio Tek Instruments, Inc. US) at 450 nm. The level of m6A methylation was calculated according to the manufacturer’s instructions.

### Co-immunoprecipitation (CoIP) assay

The CoIP assay was performed using Protein A/G PLUS-Agarose (sc-2003; Santa Cruz) according to the manufacturer’s instructions. Briefly, pre-clear grouped samples by incubating with a control immunoglobulin G (IgG) and protein A/G beads, add the specific antibody against the target protein to the pre-cleared sample and incubate it overnight at a low temperature (usually 4 °C) on a rocking platform. Elute the proteins from the beads by adding an elution buffer or heating the sample under denaturing conditions, to analyze the immune-precipitated proteins by western blotting.

### Survival analysis and treatment response evaluation

Many online data bases were applied for clinical parameters analysis, and in brief, the KM-plotter, the ROC-Plot, The CBIOPROTAL, and the Star-Base V2.0 were used for practice and generating part of the results. In detail, every method was indicated in sections of results and in figure legends, for better understanding and relative reference. More specifically, the heat-map of PAN-CANCER ANALYSIS was generated by using CBIOPROTAL at section of gene CNA/AMPLIFICATION/MUTATION section [27, 28]. The Kaplan Meier plotter is capable to assess the correlation between the expression of mRNAs and proteins and survival in lung cancer samples, based on meta-analysis based discovery and validation of survival biomarkers [29, 30]. The TNM-plotter helps to compare gene expression between tumor, normal, and metastatic samples with transcriptome-level gene expression data [31]. The ROC-plotter is capable to link gene expression and response to therapy using transcriptome-level data of kinds of cancer tumor tissues [32, 33].

### Statistical analysis

Statistical analysis was carried out by using Graph Pad Prism 8 and SPSS 20.0 software (SPSS Inc., Chicago, IL, USA). All numerical data were expressed as mean ± standard deviation (SD). Experiments were carried out with three or more replicates. Two or more groups were assessed by using Student’s t test or ANOVA individually as well as Kruskal Wallis Test, Mann–Whitney Test. P < 0.05 was considered to be statistically significant.

### Cell culture, materials and agents, CCK-8 assay, sphere formation assay, Quantitative real-time PCR, western blot, circRNA microarray and m6A-circRNA epitranscriptomic microarray

See the Supplemental Experimental Procedures.

## Results

### Wnt signaling activation in lung adenocarcinoma led to poorer survival outcomes and TKI treatment resistance

**Fig. 1:**
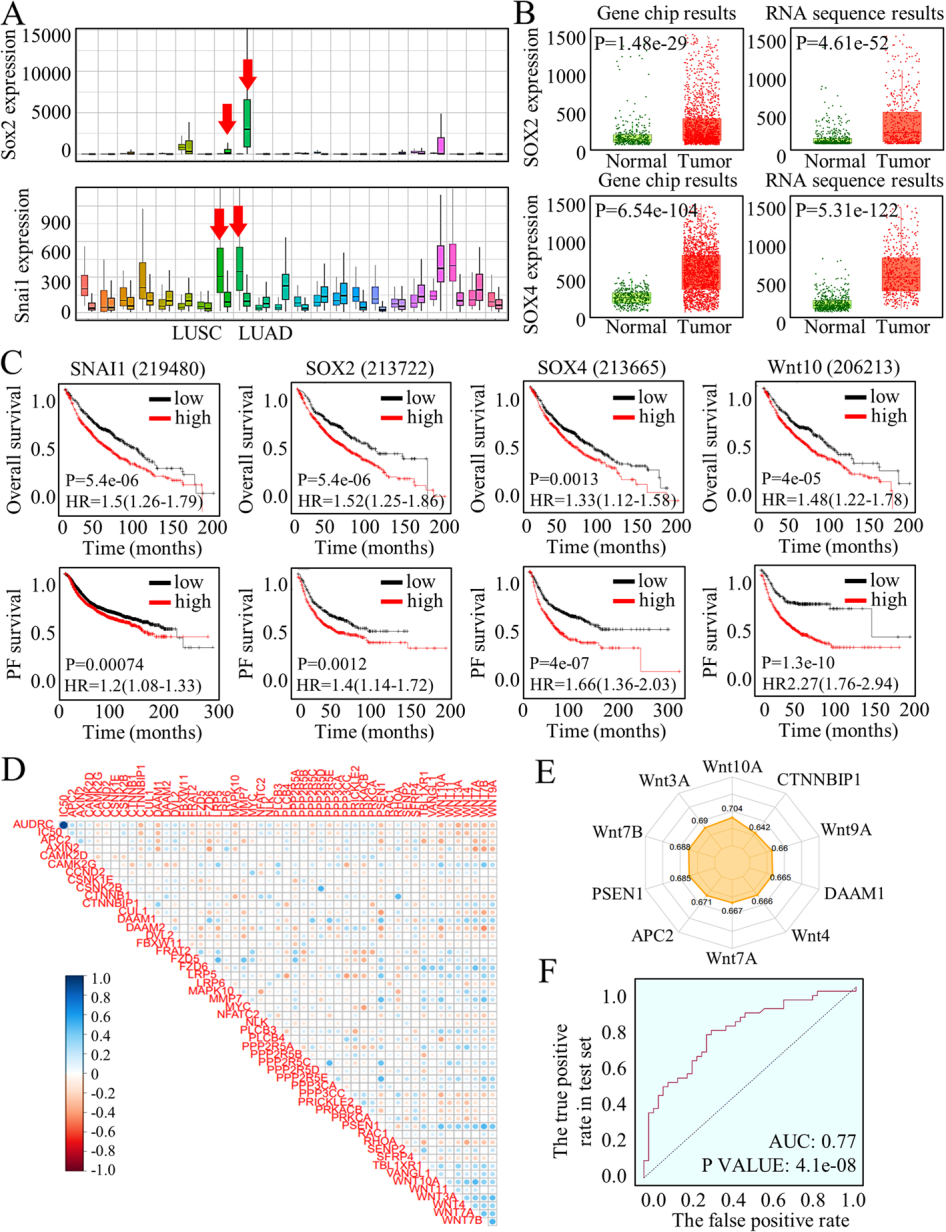
Signatures of Wnt signaling expressing status and their clinical relevance. **A** The SOX family and Snail, which are critical effectors of the Wnt signaling pathway with transcriptional and pluripotency promotion ability, exhibit significantly abnormal expression patterns. **B** RNA array and chip sequencing techniques were employed to analyze the expression patterns of the SOX family. **C** Survival analysis of Wnt signaling family factors demonstrates their negative roles in predicting overall survival and progression-free survival. **D** KEGG pathway evaluation and ROC-Plotter associated Dep-map calculation were performed to assess the effects of Osimertinib treatment. **E** The most differentially expressed functional factors and ligands were presented. **F** By clustering Wnt signaling genes, it was observed that abnormal activation of the Wnt pathway correlates significantly with diverse treatment responses and long-term therapeutic outcomes

The crucial Wnt signaling effectors were adjusted for expression patterns analysis through a screening database of Lung Adenocarcinoma (TCGA, Cohort data from Firehose Legacy) [28]. The dysregulated pathway factors were embedded in a heatmap in the form of RNA Seq V2 RSEM style (Fig. S1A) or a form of RNA Seq V2 pattern (Fig. S1B). Many of Wnt signaling factors are dysregulated, and specifically overexpressed in lung cancer tissues, among which as were shown, SOX family and Snail, the critical Wnt signaling effectors with transcriptional and pluripotency promotion ability, exhibited the most significantly abnormal expression status (Fig. 1A). In detail, with using RNA array and chip sequence, SOX family were tested for their abnormal expression patterns (Fig. 1B, Fig. S2A-D). Survival analysis of Wnt signaling family factors indicated their negative roles in predicting overall survival and in progression free survival (Fig. 1C, Fig. S2E-G), and also, the difference of mutation counts between specimens harboring either SOX2 or SNAI1 abnormities were nearly ZERO (Kruskal Wallis Test, *p* = 0.889), indicating their similar and significant roles of stimulating genes mutations (Fig. S2H).

Moreover, considering that SOX2 and SNAI1 are crucial factors for sustaining the stem cells’ renewal, we hypothesized that the stem cells’ number and renewal caused by Wnt signaling activation would be critical for the consequent therapy resistance. To initially test the Wnt signaling functions in assessing Osimertinib treatment effects, we applied KEGG pathway evaluation in ROC-Plotter associated Dep-map calculation [34], and the results was showed in Fig. 1D. The response was concluded based on the lower and upper tertiles of AUC. The significantly expressed Wnt factors were listed in Supplemental Table 2 (Sheet 1 and 2), referring to the text labels of Fig. 1D, with most differentially expressed functional factors and ligands listed in Fig. 1E. We applied the cluster of Wnt signaling genes in predicting the TKI treatment response, and the abnormal activation of Wnt pathway significantly correlated with diverse responses and long-term therapeutic outcomes (Fig. 1F). The forest model data was listed in Sheet 3 of Table S2, and Wnt signaling deeply affected the Osimertinib treatment effects. The entire row data referring to Osimertinib treatment were listed in Supplemental Table 3.

### The active Wnt signaling maintained stem cells’ renewal and contributed to TKI resistance

Within malignancies, a small, dormant group of stem cells maintains a consistent silence until triggered, unleashing their immense reproductive potential. Our research has revealed that the resistant H1975OR cells and HCC827OR cells contain a higher number of stem cells compared to adjacent normal cancer cells [2]. Consequently, we embarked on a quest to identify potential markers that could accurately distinguish this group of stem cells.

Initially, we explored the baseline settings using CD44 + , CD44 + /133 + , and CD44^+^/CD24^−/low^ subpopulations. Unfortunately, none of these markers were able to precisely and accurately separate the stem cells. To provide further details, the proportions of CD44^+^, CD44^+^/133^+^, and CD44^+^/CD24^−/low^ cells accounted for 53%, 25%, and 71%, respectively.

**Fig. 2:**
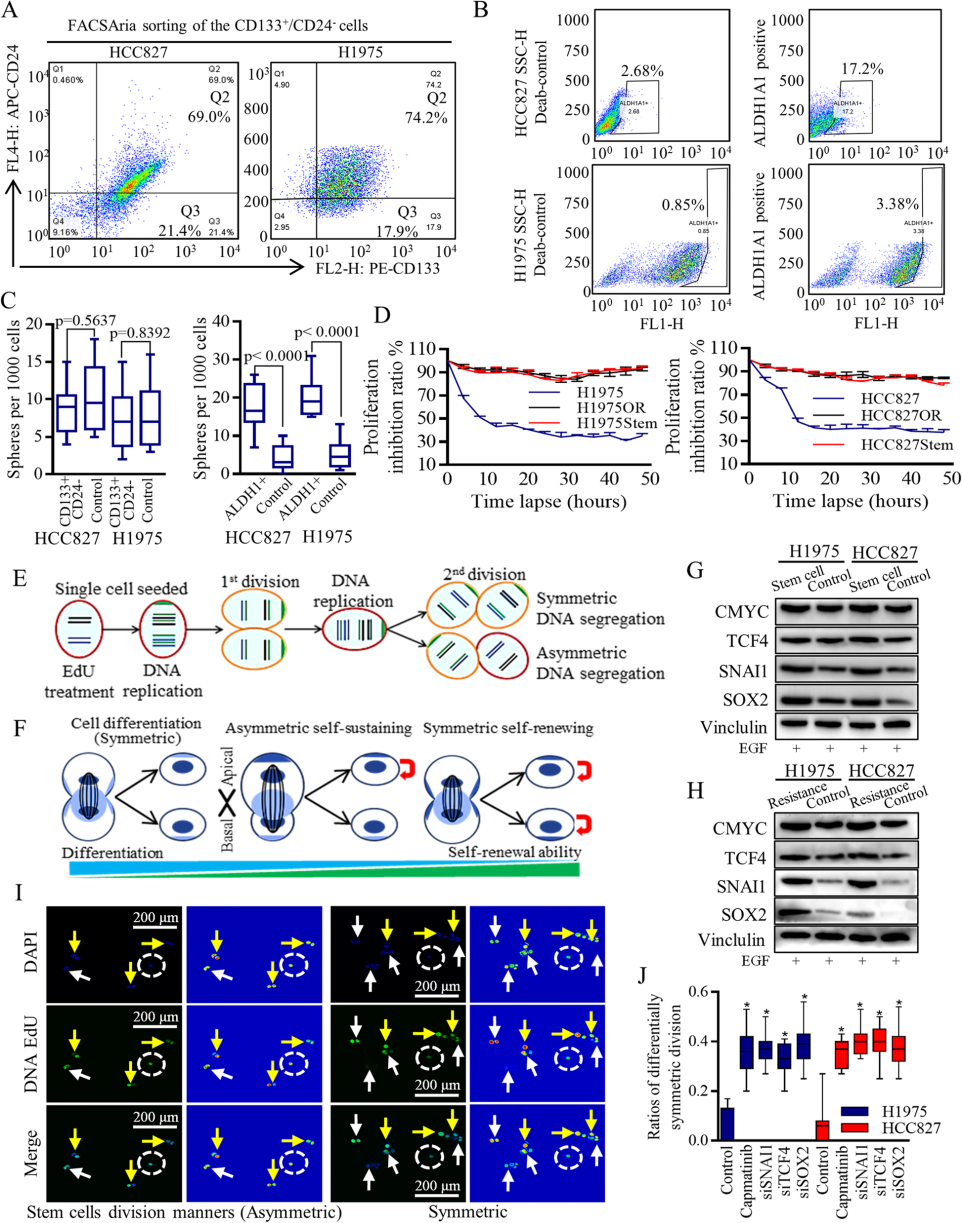
Stem-like cells retained the regeneration capability to re-produce lung cancer group in a Wnt activation dependent manner. Different markers referring to identifying the Stem-like cells may distinguish diverse sub-group of different signatures. **A** In HCC827 cell lines and H1975 cell lines, the groups of stem-like cells with CD133^+^/CD24^−^ markers was 21.4% and 17.2% relatively. **B** The stem cells of ALDH1A1^+^ surface marker accounted for a small group of 2.34% (HCC827) and 1.31% (H1975) respectively. **C** the ALDH1A1^+^ cells could renew themselves and form the spheres effectively. **D** The amplified stem cells population of spheres were naturally resistant to Osimertinib treatment, as was the Osimertinib resistant lung cancer cells. **E**–**F** Schematic images illustrated the different cells division manners, and the unpaired DNA segregation modes. The palette color set of rainbow mode was used to show the glow scale of each single cell at the dividing process. Wnt signaling effectors were overexpressed in lung cancer stem cells (**G**), and in the resistant cells (**H**). **I**-**J** Wnt signaling functions were verified to be effectively repressed by Capmatinib, TCF-4, SNAI1, SOX2 inhibition, and then was found to promote the differentiability-oriented symmetrical cell dividing, and decreased the asymmetrical self-renewing and the symmetrical self-renewing

In the case of HCC827 cell lines and H1975 cell lines, we were able to identify smaller groups of stem-like cells that expressed the CD133^+^/CD24^−^ markers (Fig. 2A, Fig. S3A, raw data in Fig. S3B-C), or the ALDH1A1^+^ marker (Fig. 2B, raw data in Fig. S4A-B). Moreover, the ALDH1A1^+^ cells demonstrated a capacity for self-renewal and efficient formation of spheres (Fig. 2C). Notably, these spheres exhibited inherent resistance to 2uM of Osimertinib treatment (Fig. 2D).

Wnt signaling activation was critical for sustaining the malignant division manners that stimulated the daughter cells’ renewal [14, 24], which could be defined as symmetry or asymmetry by using DNA staining [11, 35] (Fig. 2E). Stem cells have the ability to generate daughter cells with different characteristics, thereby influencing the expansion, maintenance, or reduction of the stem cell population through various mechanisms. This process of cell division can be categorized into symmetrical cell differentiation, symmetrical self-renewal, and asymmetrical self-renewal, as depicted in Fig. 2F. In our investigation, we examined and confirmed the overexpression of Wnt signaling effectors in lung cancer stem cells (Fig. 2G), as well as in the resistant cells (Fig. 2H). To repress Wnt functionality, we employed Capmatinib, TCF-4, SNAI1, and SOX2 inhibition methods [14, 16, 24] promoted the symmetrical cell differentiation, and decreased the asymmetrical self-renewing and the symmetrical self-renewing (Fig. 2I-J, Fig. S5A, B-C), abolishing sphere-forming ability greatly (Fig. S5D).

### The signatures of dysregulated circRNAs in TKI resistant lung cancer cells

**Fig. 3:**
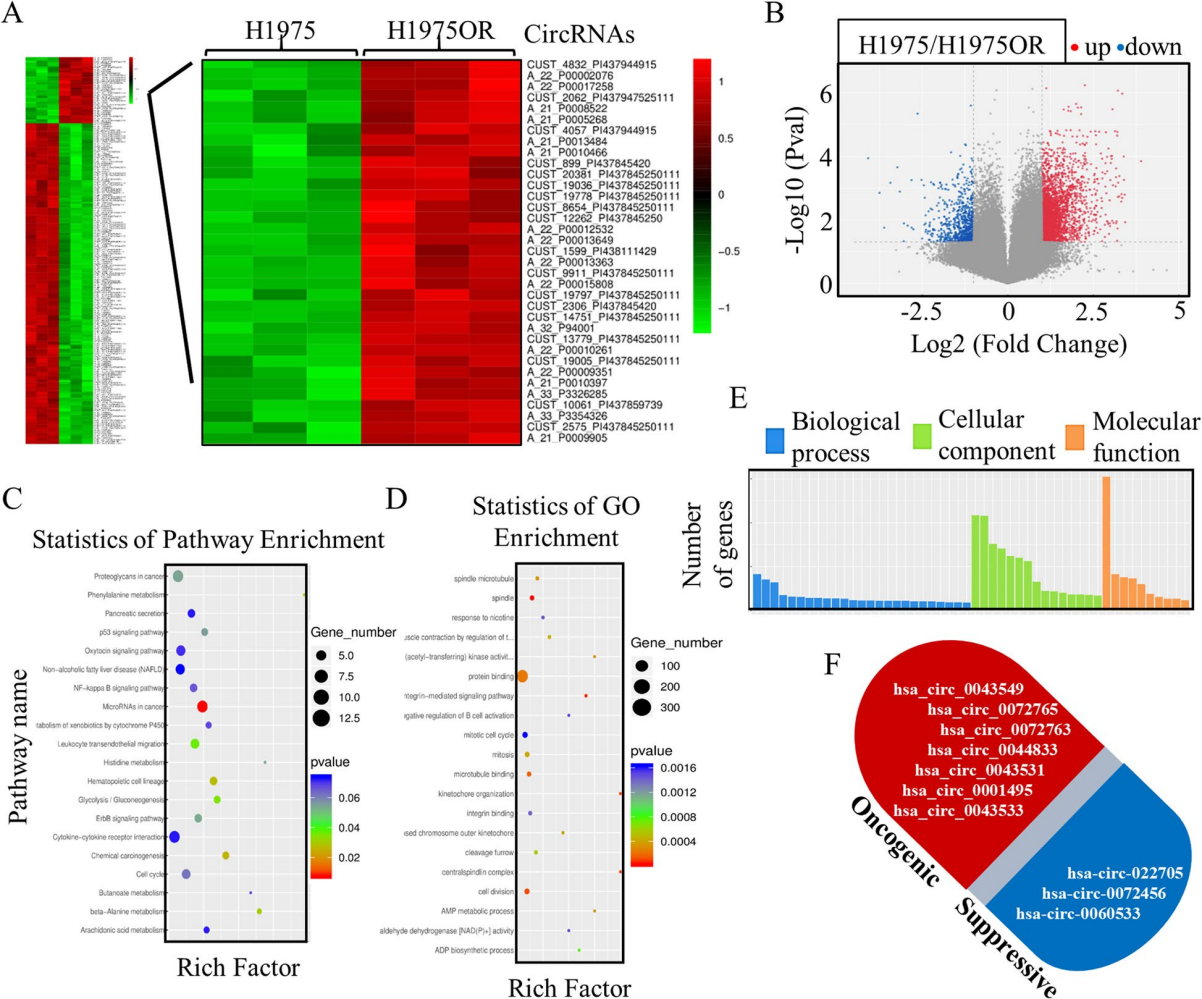
CircRNAs participated in the Osimertinib therapy resistance. **A**-**B** The ribosomal RNA-depleted total RNAs of the resistant HCC827OR cells and H1975OR cells were adjusted to perform the RNA-seq, and the differentially expressed circRNAs were preliminary screening. The informatic analysis of cellular and signaling functions indicated the differentially expressed circRNAs were possibly participated in microRNAs functions (**C**), in cell division (**D**), in molecular functions, and in cellular components (**E**). **F** The candidate circRNAs with significant dysregulations and potential abnormal functions were laid out

To generate a circRNAs profiling database and to test their translation probability, we performed the RNA-seq analysis of ribosomal RNA-depleted total RNA of the resistant HCC827OR cells and H1975OR cells (Fig. 3A), and the differentially expressed circRNAs with significance were illustrated in volcano plots (Fig. 3B). Multiple functional analysis was performed initially, and the differentially expressed circRNAs mainly participated in microRNAs functions (Fig. 3C) and in cell division (Fig. 3D), which were closely related to mechanical processing that contributed to therapy resistance. We also noticed the disrupted molecular functions and cellular components driven by the dysregulated circRNAs (Fig. 3E), and the candidate circRNAs with significant dysregulations, and potential abnormal functions were drafted in Fig. 3F.

### M6A promotion of circ-FBXW7 expression was critical for stem cells’ renewal inhibition

**Fig. 4:**
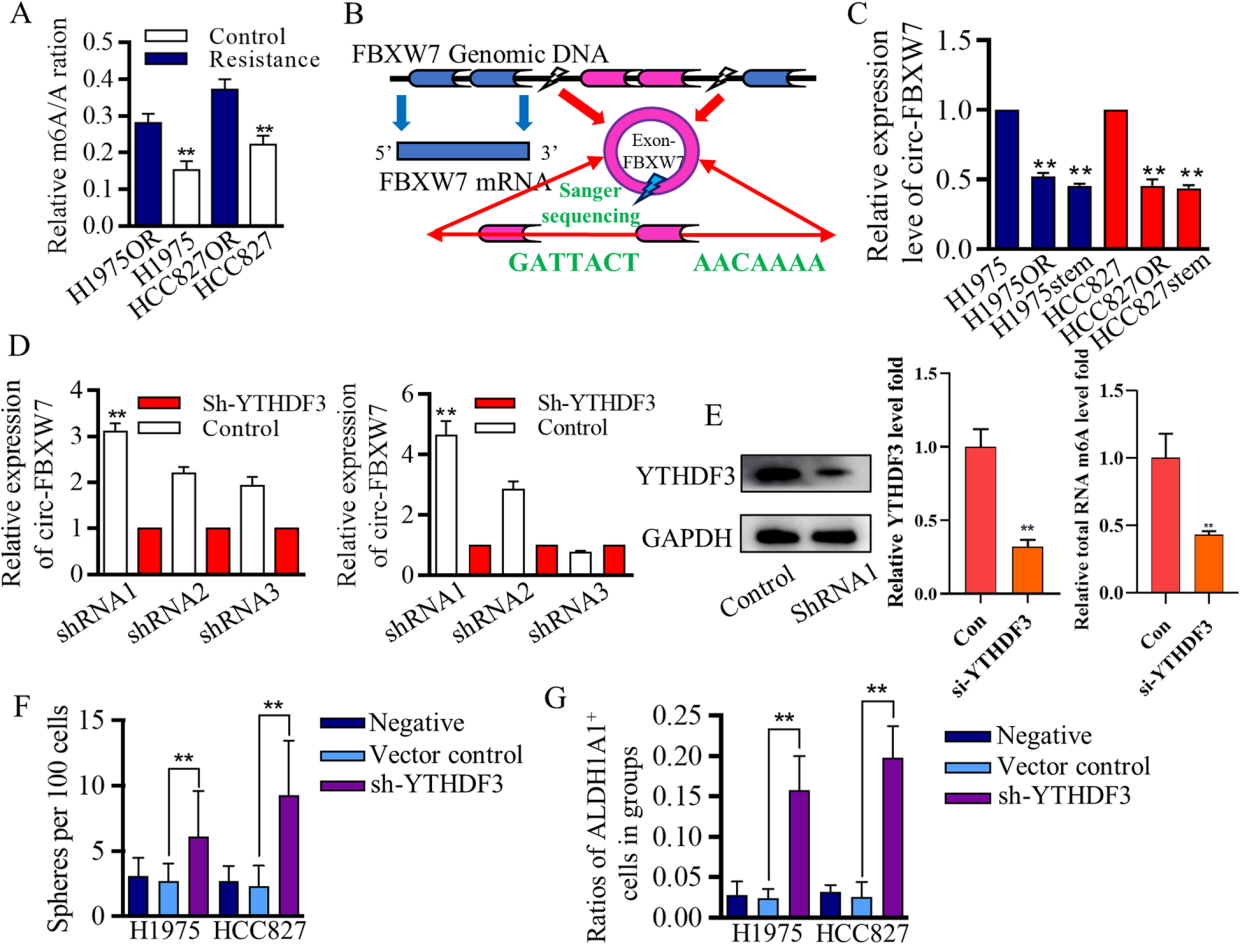
m6A modified circ-FBXW7 expression in resistant lung cancer cells **A** The m6A levels of total RNAs from resistant cells were more abundant than that of sensitive original cells. **B** The differentially expressed hsa-circ-0001451 (circ-FBXW7) was matched in circRNA Database and the structure was illustrated for mechanistic understanding. **C** circ-FBXW7 was lowly expressed in Osimertinib resistant cells and in stem-like cells. **D**-**F** The lentiviral based YTHDF3 knock-down systems inhibited its expression significantly, and LC–MS/MS tests revealed the YTHDF3 associated m6A levels. **F**-**G** YTHDF3 inhibition decreased the m6A modification and therefore promoted the stem cells renewal that identified by spheres forming assay and surface marker of ALDH1A1

Aberrant m6A status was reported to be associated with abnormal mRNAs expressions, and molecular dysfunctions [2, 19, 36]. To explore the m6A-related therapy resistance, we first identified that the m6A levels of total RNAs from resistant cells were more abundant than that of sensitive original cells (Fig. 4A), strongly indicating the m6A involvement of therapy resistance. Generating circRNAs could be controlled by multiple procedures, and m6A stimulated the RNA circling process [17, 36]. Array-star sequencing was applied for detecting m6A differences referring to every single circRNA between stem cells and adjacent cancer cells (Fig. S6A), and m6A signatures were drafted for following candidates' (Fig. S6B). The irregulated circRNAs with strong m6A signatures were compared with the screening results of Fig. 3A section, and among these specific candidates, hsa-circ-0001451, which was formed by circularization of exon 3 and exon 4 of the FBXW7 gene, was chosen for its significant m6A difference and its potentially vital suppressive roles in cancer mediation we previously identified [15, 37]. The differentially expressed hsa-circ-0001451 (circ-FBXW7) was matched in circRNA Database (Fig. 4B), and its expression patterns in resistant cells were assessed (Fig. 4C). Array-star sequencing and Gene-ID identification analysis showed the lower m6A level at circ-FBXW7 of Osimertinib resistant cells (Fig. S6C-D).

**Fig. 5:**
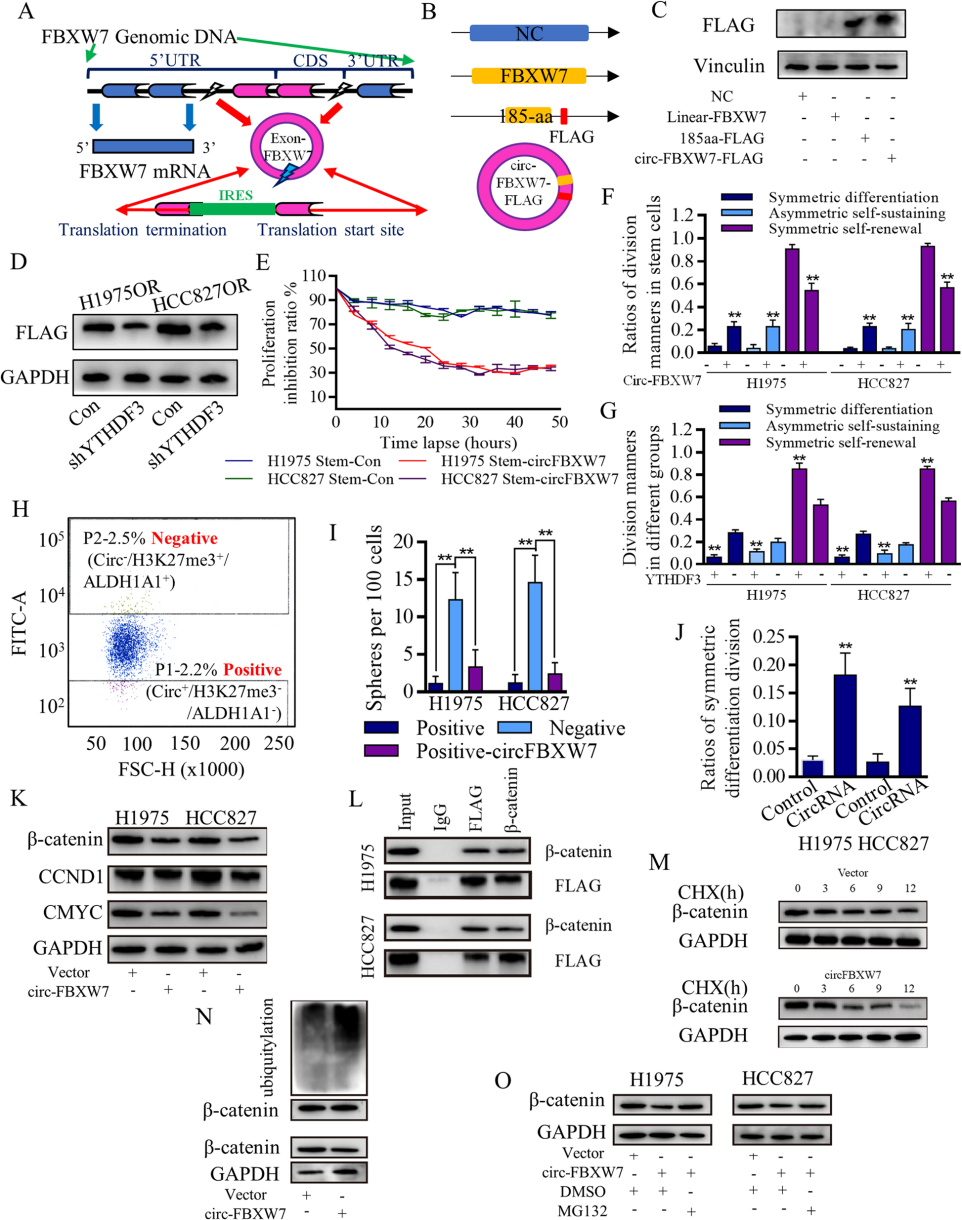
Translated circ-FBXW7 suppressed stem cells’ renewal in a β-catenin ubiquitylation way. **A** Schematic image illustrated the IRES independent translation potential of circ-FBXW7. **B** Designation of junction probe and Flag-tag to detect translated circ-FBXW7-AA. **C** The specifically designed and synthesized FLAG antibody successfully detected circ-FBXW7-AA in 293 T cells. **D** The reader of YTHDF3 inhibition blocked m6A quantity, and decreased circ-FBXW7-AA level. Over-expressed circ-FBXW7 inhibited the proliferation of stem cells group (**E**), and stimulated the differentiated-orientation cells dividing (**F**) The tags of “negative” and “positive” are referring to marker of circ-FBXW7 solely, and the positive-circFBXW7 was set as the positive control. **G** The increased differentiated-orientation cells dividing, together with the increased asymmetric cells dividing, decreased the malignant behaviors. Similarly, YTHDF3 inhibition failed to stimulate the circ-FBXW7 dependent differentiated cells division, and helped to restore the symmetric cells dividing. **H** The selecting cells of FSC gate was grouped based on distribution patterns of phenotypic markers of circ-FBXW7/H3K27me3/ALDH1A1. **I** The cells with negative label (circ-FBXW7^−^/H3K27me3^+^/ALDH1A1^+^) showed much higher ability to form the spheres when assessing in per 100 cells, and the positive label (circ-FBXW7^+^/H3K27me3^−^/ALDH1A1^−^) greatly decreased the spheres forming ability. **J** Enforced circ-FBXW7 stimulated symmetric differentiation dividing, and consequently resulted in spheres forming inhibition. **K** The overexpressed circ-FBXW7 affected the downstream Wnt/β-catenin signaling (CCND1/CMYC) to conduct the renewal inhibition effects. **L** Co-immunoprecipitation assay revealed the endogenous circ-FBXW7-185AA could interact with endogenous β-catenin in both HCC827 cells and H1975 cells. Circ-FBXW7-185AA negatively regulated the protein stability of β-catenin (M), the ubiquitination status of β-catenin increased with overexpressed circ-FBXW7-185AA (N). **O** The ubiquitination level of β-catenin gradually increased with the overexpressed circ-FBXW7-185AA, and MG132 weakened the inhibitory effect of circ-FBXW7-185AA on β-catenin expression

To further characterize the uncertain roles of m6A in generating the resistance, we used siRNAs to tentatively test the m6A associating processers that may control circ-FBXW7 genesis, and found that YTHDF3 strongly affected circ-FBXW7 level (Fig. S6E), which were further confirmed by using the lentiviral-based YTHDF3 knock-down systems (Fig. 4D), which were confirmed by blotting and LC–MS/MS tests (Fig. 5E). To verify the m6A controlling of stem cells, we found that YTHDF3 inhibition promoted the stem cells proliferation (Fig. S6F), and these results logistically proved that functional cascade of YTHDF3/m6A/circFBXW7.

### The m6A stimulation of circ-FBXW7-AA translation determined the stem cells differentiation

Circ-FBXW7 was identified with junction a probe to test the ORF and internal ribosomal entrance site (IRES) (Fig. 5A), which would conduct and initiate the 5’-cap independent translation [17, 38, 39]. Circ-FBXW7 overexpression was identified (Fig. S7A) and was verified with upregulated circ-FBXW7-AA using specifically designed FLAG antibody (Fig. 5B) of the plasmids in 293 T cells (Fig. 5C). We found that m6A affected the translation efficiency of circ-FBXW7-185AA, and specifically, YTHDF3 determined both the circRNA expression and the AA-peptide level (Fig. 5D), the reader of YTHDF3 inhibition significantly decreased the circ-FBXW7-185aa expression. Different dividing manners of H1975, HCC827 cells, and H1975OR, HCC827OR cells were identified as symmetrical differentiation, symmetric renewal, and asymmetric division. Over-expressed circ-FBXW7 inhibited the proliferation of the stem cells group (Fig. 5E). It stimulated the differentiated-orientation cells dividing (Fig. 5F). The symmetrical dividing cells could be identified as differentially (Fig. S7B) and renewably (Fig. S7C), and the equal distribution of both ALDH1A1 and H3K27me3 were captured. The unequal distribution of CBP, H3K27ac, and NUMB help to identify the asymmetrical dividing cells (Fig. S7D). Similarly, YTHDF3 inhibition failed to stimulate the circ-FBXW7 dependent differentiated cells division (Fig. 5G).

The unpaired distribution of tumor suppressive and oncogenic genes finally led to daughters of diverse signatures, and to prove the different signatures of daughter cells, FACSAria-based single cell sorting was applied to acquire and seed the single dividing daughter cell. The SSC gate threshold was set, as was shown in Fig. S7E, and the selecting cells of FSC gate were grouped based on distribution patterns of phenotypic markers of circ-FBXW7/H3K27me3/ALDH1A1 (Fig. 5H). The cells with negative circ-FBXW7 (labeled as “negative”, and the whole term should be circ^−^/H3K27me3^+^/ALDH1A1^+^), which accounted for the renewable asymmetric dividing cell, showed much higher ability to form the spheres when assessing in per 100 cells (not as usual as per 1000 cells) (Fig. 5I). The positive groups with circ-FBXW7^+^/H3K27me3^−^/ALDH1A1^−^ phenotypic markers greatly decreased the spheres forming ability when comparing to the positive control group and the cells with negative circ-FBXW7 and positive H3K27me3/ALDH1A1 markers. Enforced circ-FBXW7 stimulated symmetric differentiation dividing (Fig. 5J), which consequently resulted in spheres forming inhibition, and affected the tumor formation (Fig. S7F-G).

The overexpressed circ-FBXW7 affected the downstream signaling of Wnt/β-catenin pathway to conduct the renewal inhibition effects (Fig. 5K), and we found that endogenous circ-FBXW7-185AA interacted with endogenous β-catenin in both HCC827 cells and H1975 cell through co-immunoprecipitation assay (Fig. 5L). The translated peptide of circ-FBXW7-185AA may regulate β-catenin in a post-translation regulating way, and we therefore ﻿performed a cycloheximide (CHX) chase assay in circ-FBXW7-overexpressed and negative control cells. Results suggested that circ-FBXW7-185AA negatively regulated the protein stability of β-catenin (Fig. 5M). The ubiquitination status of β-catenin plays an essential role in the stability of Wnt/β-catenin signaling functions, to date, we examined the ubiquitination status of β-catenin with overexpressed circ-FBXW7-185AA (Fig. 5N). The ubiquitination level of β-catenin gradually increased with the overexpressed circ-FBXW7-185AA, and the addition of MG132 again weakened the inhibitory effect of circ-FBXW7-185AA on β-catenin expression (Fig. 5O).

### Circ-FBXW7 repressed Wnt/β-catenin signaling activation and therefore released Let-7d to form the positive signaling loop

**Fig. 6:**
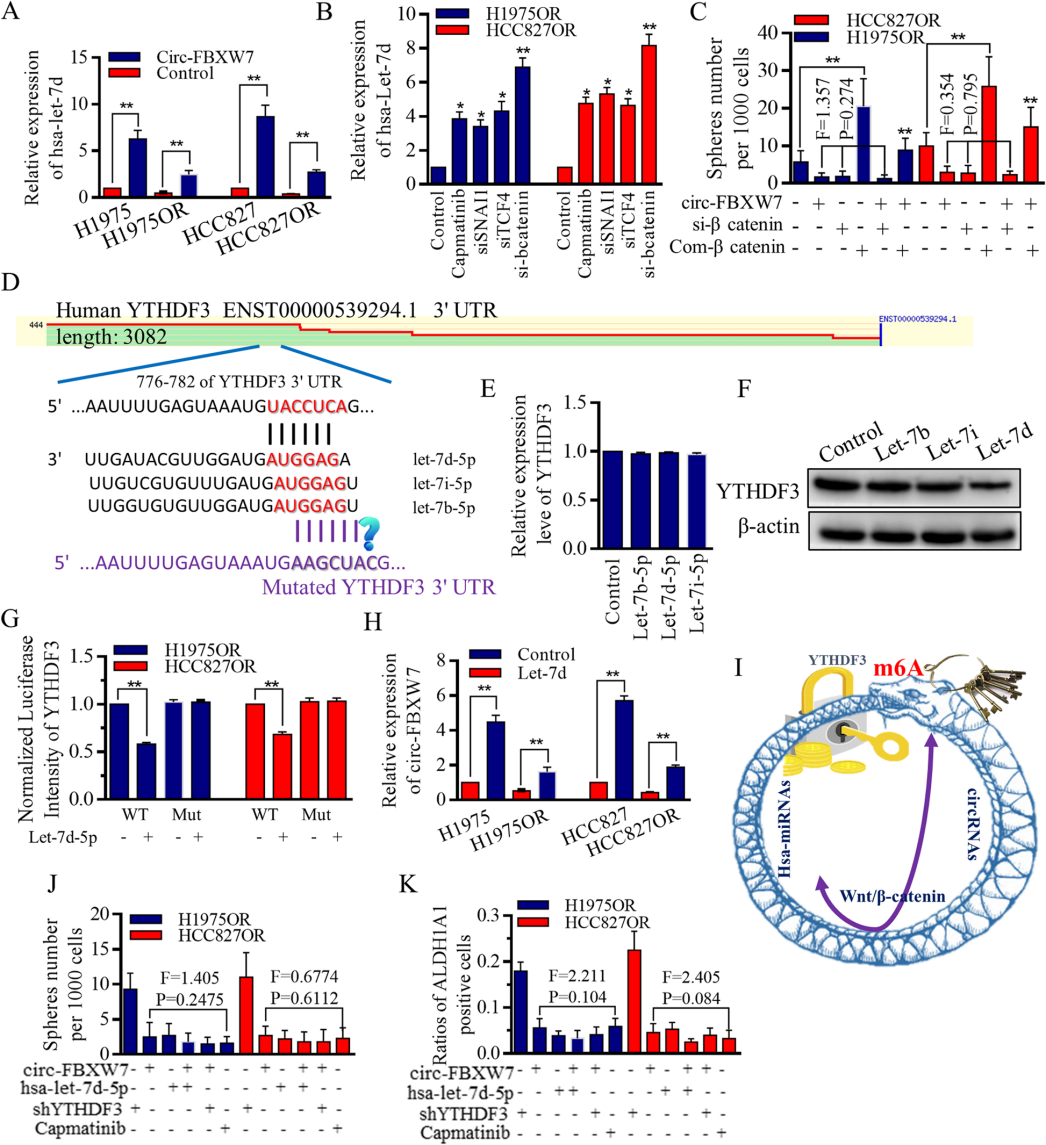
Circ-FBXW7 did not function through Let-7 rescue, but formed negative feedback through Wnt/β-catenin signaling mediation. **A** The overexpression of circ-FBXW7 led to an upregulation of Let-7d expression. **B** In Osimertinib-resistant HCC827OR and H1975OR cells, inhibition of the Wnt signaling pathway resulted in increased Let-7d expression. Strong stimulation of Let-7d expression was observed upon β-catenin inhibition. **C** The effects of Wnt signaling on sphere-forming ability were assessed, and no significant differences were observed among groups treated with circ-FBXW7, si-β-catenin, or their combination, indicating their similar functions. However, the synthesized compound of Wnt signaling stimulator increased sphere-forming ability, which was partially relieved by circ-FBXW7. **D** Putative binding sites were identified between the m6A readers (YTHDF3) and Let-7 miRNAs. **E** Manipulation of Let-7b, Let-7d, or Let-7i did not significantly alter the mRNA levels of YTHDF3. **F** Among the Let-7 miRNAs tested, Let-7d showed the most effective decrease in YTHDF3 expression. **G** Let-7d inhibited the luciferase activity of wild-type YTHDF3 mRNA, but had no effect on mutant groups. **H** Let-7d inhibited circ-FBXW7 expression in an YTHDF3 repression-dependent manner. **I** Let-7d and circ-FBXW7 formed a negative feedback loop. **J** Enforcing circ-FBXW7, Let-7d, or inhibiting YTHDF3 all decreased the self-renewal ability of resistant H1975OR and HCC827OR cells, with Capmatinib used as a positive control. **K** Upregulation of circ-FBXW7 and Let-7d reduced the stem cell population, while inhibition of YTHDF3 increased stem cells expressing the ALDH1A1 surface marker

It is reported that Wnt/β-catenin pathway could depress Let-7 maturation and expression through transcriptionally activating the LIN28 [40–42], and we previously identified the suppressive roles of FBXW7 exerted on Wnt signaling functions [15, 37]. We hypothesized that the possible connection between circ-FBXW7 and let-7 family of miRNAs may be of great value in stem cells’ renewal and therapy resistance. We first confirmed that circ-FBXW7 stimulated Let-7d significantly (Fig. 6A), and the Wnt signaling inhibition in Osimertinib resistant HCC827OR and H1975OR cells released Let-7d expression incredibly (Fig. 6B). Among all the inhibitive factors, β-catenin inhibition strongly stimulated Let-7d expression (Fig. 6B), as previously reported, and moreover, the differences among groups receiving either circ-FBXW7, si-β-catenin, or their combination, are not noticeable, indicating their similar functions (Fig. 6C).

To further elucidate the mechanistic regulations of Let-7d that may conduct through, we screened most of the relevant databases, and found the putative binding sites among the m6A readers of YTHDF3, and Let-7 miRNAs (Fig. 6D). YTHDF3 mRNA did not change significantly when manipulating either Let-7b/d/i (Fig. 6E), but the protein level decreased greatly when Let-7d was introduced (Fig. 6F). Let-7d inhibited the luciferase activity of wild-type YTHDF3 mRNA, but not functioned in mutant groups (Fig. 6G). m6A controlled the circ-FBXW7 expression, and our results testified the theory that enforcing Let-7d could inhibit circ-FBXW7 expression in a YTHDF3 repression dependent way, forming a negative feedback loop (Fig. 6H), and the speculated cascade was drawn in Fig. 6I. Functionally, either enforcing circ-FBXW7, enforcing Let-7d, or inhibiting YTHDF3, could all decrease the self-renewal ability of resistant H1975OR cells and HCC827OR cells (Fig. 6J), and shrink the stem cells group with ALDH1A1 surface marker (Fig. 6K).

### Clinical significances of m6A regulators and let-7 in TKI treatment response

We conducted a comprehensive analysis of available datasets to evaluate the potential of various factors in predicting the response to TKI treatments, specifically focusing on Let-7 and m6A-related proteins. Let-7 demonstrated high sensitivity and specificity in predicting therapy response, as indicated by the analysis of multiple datasets (Fig. S8A, Supplemental Table 4, sheet1). Additionally, YTHDF3 showed advanced accuracy in sensitivity and specificity in predicting TKI treatment efficacy across different datasets, including Depmap (Fig. S8B, Supplemental Table 4, sheet2), GDSC1 (Fig. S8C, Supplemental Table 4, sheet3), and CTRP (Supplemental Table 4, sheet4), highlighting its crucial role in mediating the effects of TKI treatments. On the other hand, YTHDF1, YTHDF2, METTL3, and METTL14 did not exhibit advanced accuracy in sensitivity and specificity (Fig. S9A-D).

We further examined the connection of these factors in clinical samples, specifically in normal tissues adjacent to lung adenocarcinoma (LUAD) and lung squamous cell carcinoma (LUSC). No significant relationship was observed, except for an inverse relationship between Let-7d and METTL3 in LUAD (Fig. S8D-G, Fig. S9E-G). However, it is important to note that METTL3 did not demonstrate accuracy in sensitivity and specificity regarding TKI treatment efficacy (Fig. S9C), which aligns with the research objectives and expectations of this study.

## Discussion

Lung cancer has emerged as a leading cause of death, persistently claiming a rising number of lives despite advancements in diagnostic approaches. While therapeutic strategies and methods have continuously improved, the mortality rate remains alarmingly high among cancer-related deaths. As the most commonly diagnosed malignancy, largely influenced by air pollution and smoking habits, lung cancer treatment has witnessed significant upgrades and advancements in recent years, particularly with the emergence of targeted therapies and immunotherapies. However, the heterogeneous nature of lung cancer, characterized by diverse hierarchical signatures, poses challenges. Within this heterogeneous group, the population of stem cells plays a critical role in tumor dormancy, therapy resistance, and long-term recurrence. Therapies designed to target rapidly proliferating cancer cells often prove ineffective against the quiescent and resilient cell subpopulation. These naturally resistant cells possess the ability to sustain and regenerate themselves even as the more susceptible cells succumb to the effects of chemotherapy and radiation. Thus, a perplexing problem arises.

The targeted therapy of EGFR tyrosine kinase inhibitors showed great advantages compared to traditional chemo-radio treatments in patients with specific and sensitive sequence mutations. Recent studies have propelled TKIs to the forefront of adjuvant therapy as the first-line approach for lung cancer patients, offering tremendous benefits and promising prospects. However, therapy resistance can emerge even after receiving these highly effective treatments, particularly in patients who have undergone third-generation TKIs or advanced therapies. This resistance often leads to rapid disease progression and leaves patients in a helpless situation. Although immune therapy holds potential in overcoming resistance, its widespread application is limited due to uncertain side effects and clinical adaptability. Therefore, methods that can sensitize TKI treatments or re-sensitize resistant cells have become a preferred strategy and hold priority when making clinical decisions.

We aimed to explore and identify potential methods and agents that could effectively reverse the resistance process in lung cancer. In previous studies, we identified candidate miRNAs, lncRNAs, and circRNAs in resistant lung cancer cells and investigated their roles in sponging-based competitive regulation, feedback axes, and m6A regulation. Notably, we found that metformin facilitated the reversal of TKI treatment effects. Our earlier research demonstrated the inhibitory effect of FBXW7 on Wnt signaling activation and stem cell renewal. Building upon this knowledge, we screened for dysregulated circRNAs in Osimertinib-resistant lung cancer groups and investigated the suppressed circ-FBXW7's role in controlling the functional EGFR pathway.

Through bioinformatic analyses, we evaluated potential downstream factors contributing to the effects of circ-FBXW7. We employed molecular biological methods such as chromatin immunoprecipitation, co-immunoprecipitation, and RNA immunoprecipitation to confirm the interplay between m6A, circ-FBXW7, EGFR, and the WNT regulatory cascade. Our results revealed the formation of a circ-FBXW7/Wnt/Let-7 feedback axis via the m6A regulator YTHDF3. Furthermore, we made a groundbreaking discovery by proving the critical roles of the stem cell group in driving the resistance process. The division manners of stem cells determined their expansion, and we observed a delicate and mutually restricted signaling pathway. Wnt signaling decreased Let-7 miRNAs, which could be rescued by circ-FBXW7. However, Let-7 reciprocally inhibited m6A, thereby maintaining circ-FBXW7 expression levels (Fig. S10). Using FACSAria-based cell sorting, we monitored the distinct distribution patterns of key stemness regulators in separate daughter cells, which determined their fates and, subsequently, the future of stem cells.

Importantly, we identified the functional role of translated circ-FBXW7-AA in regulating the division patterns of stem cells and controlling their population expansion. Our findings revealed the intricate cascade of the circ-FBXW7/Wnt/Let-7 feedback axis, which is dependent on the m6A connection. This study represents an unprecedented and innovative exploration of the underlying mechanisms involved in therapy resistance and stem cell regulation in lung cancer.

## Conclusion

Our findings emphasize the significant role of translatable circRNA in determining the response to TKI therapy, revealing the regulatory signaling cascade and stemness regulation underlying this process. Importantly, we highlight the pivotal contribution of the stem cell population in driving therapy resistance, with m6A playing a crucial role in maintaining the resistant state by influencing the circularization and translation of circRNA within the stem cell group. This mechanism effectively pushes the continuously renewing stem cells towards the forefront of clinical treatments, representing a previously unrecognized aspect of therapy resistance.

## Supplementary Information

**Additional file 1:** **Supplemental Figure 1. **Heatmap results introducing the genes screening of Wnt signaling activation status.

**Additional file 2:** **Supplemental Figure 2. **Wnt signaling members expressing and clinical signatures.

**Additional file 3:** **Supplemental Figure 3. **Raw images referring to flow analysis.

**Additional file 4:** **Supplemental Figure 4. **Raw images referring to flow analysis.

**Additional file 5:** **Supplemental Figure 5. **Wnt signaling status affected the stem cells’ division.

**Additional file 6:** **Supplemental Figure 6. **M6A regulation of hsa-circ-0001451 (circ-FBXW7) affected the stem cells’ renewal and the consequent therapy resistance.

**Additional file 7:** **Supplemental Figure 7. **Circ-FBXW7 controlled the cells’ division manners.

**Additional file 8:** **Supplemental Figure 8. **The correlation between let-7 family of miRNAs and YTHDF3 mRNA expression.

**Additional file 9:** **Supplemental Figure 9. **The correlation between let-7 family of miRNAs and YTHDF3 mRNA expression.

**Additional file 10:** **Supplemental Figure 10. **M6A participation in circRNAs’ dysregulation and translation determined the stem cells’ therapy response

**Additional file 11:** **Supplemental Figure 11. **Supporting information for flow analysis.

**Additional file 12:** **Supplemental Figure 12. **The original images of IF experiments referring to Fig. 2.

**Additional file 13:** **Supplemental Figure 13. **The original images of IF experiments referring to Figure S4.

**Additional file 14:** **Supplemental Figure 14. **The original images of IF experiments referring to Figure S6.

**Additional file 15.** 

**Additional file 16.** 

**Additional file 17.** 

**Additional file 18.** 

## Data Availability

The data and supporting materials regarding the findings of this study are accessible in the main document as well as in supplemental tables, figures, and additional information. Raw data associated with the images can be found in the supplemental information. However, detailed key data are not available for sharing but can be obtained from the corresponding author upon reasonable request. Data from the GSE dataset and public clinical dataset are available as specified in the “Methods and Materials” section.
