# Supplementary figures and images for: Enhancement of TKI sensitivity in lung adenocarcinoma through m6A-dependent translational repression of Wnt signaling by circ-FBXW7
Source: Mol Cancer. 2023 Jul 1;22:103. doi: 10.1186/s12943-023-01811-0 (PMC10314519; doi:10.1186/s12943-023-01811-0)

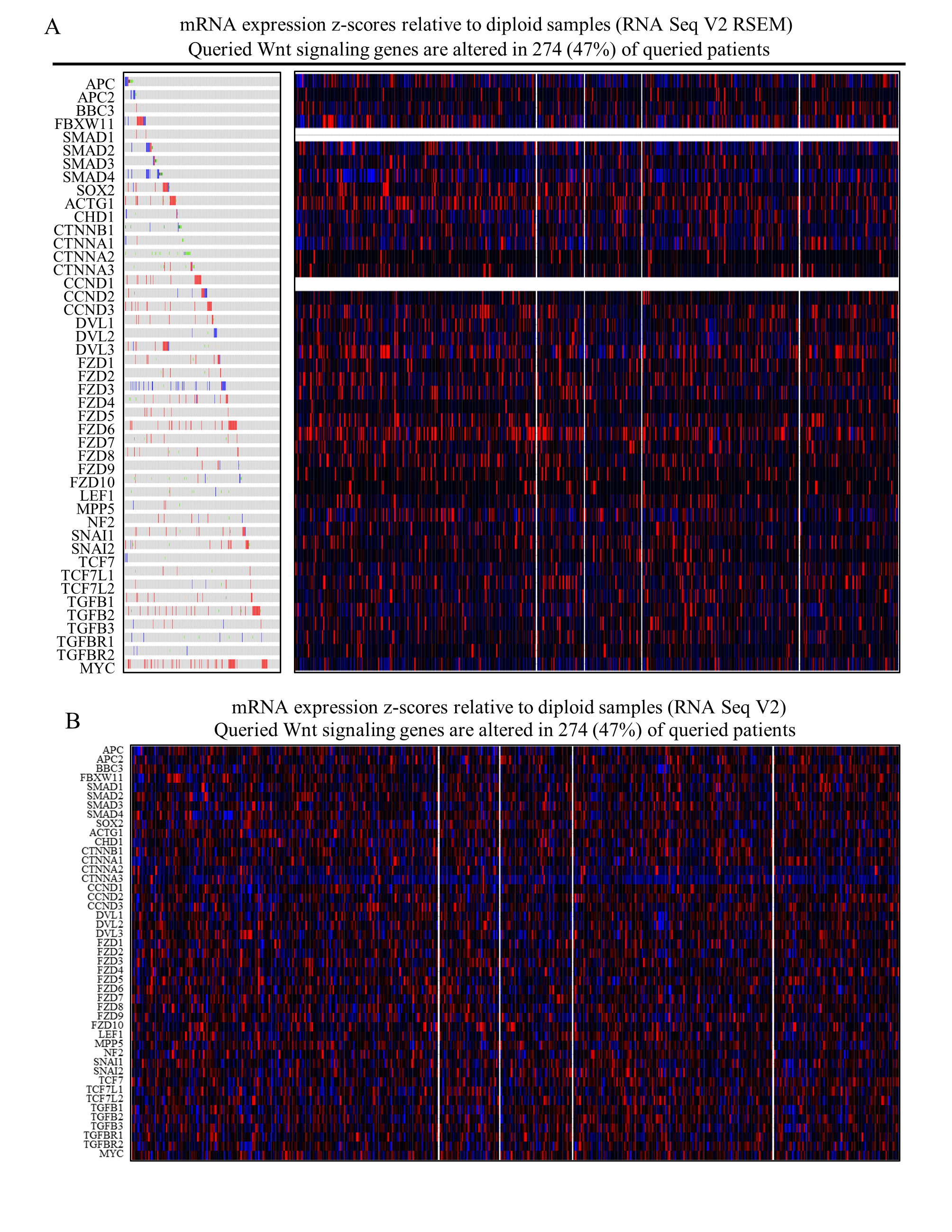

Supplement: Supplementary file 1 — Additional file 1: Supplemental Figure 1. Heatmap results introducing the genes screening of Wnt signaling activation status. [file 12943_2023_1811_MOESM1_ESM.tif]

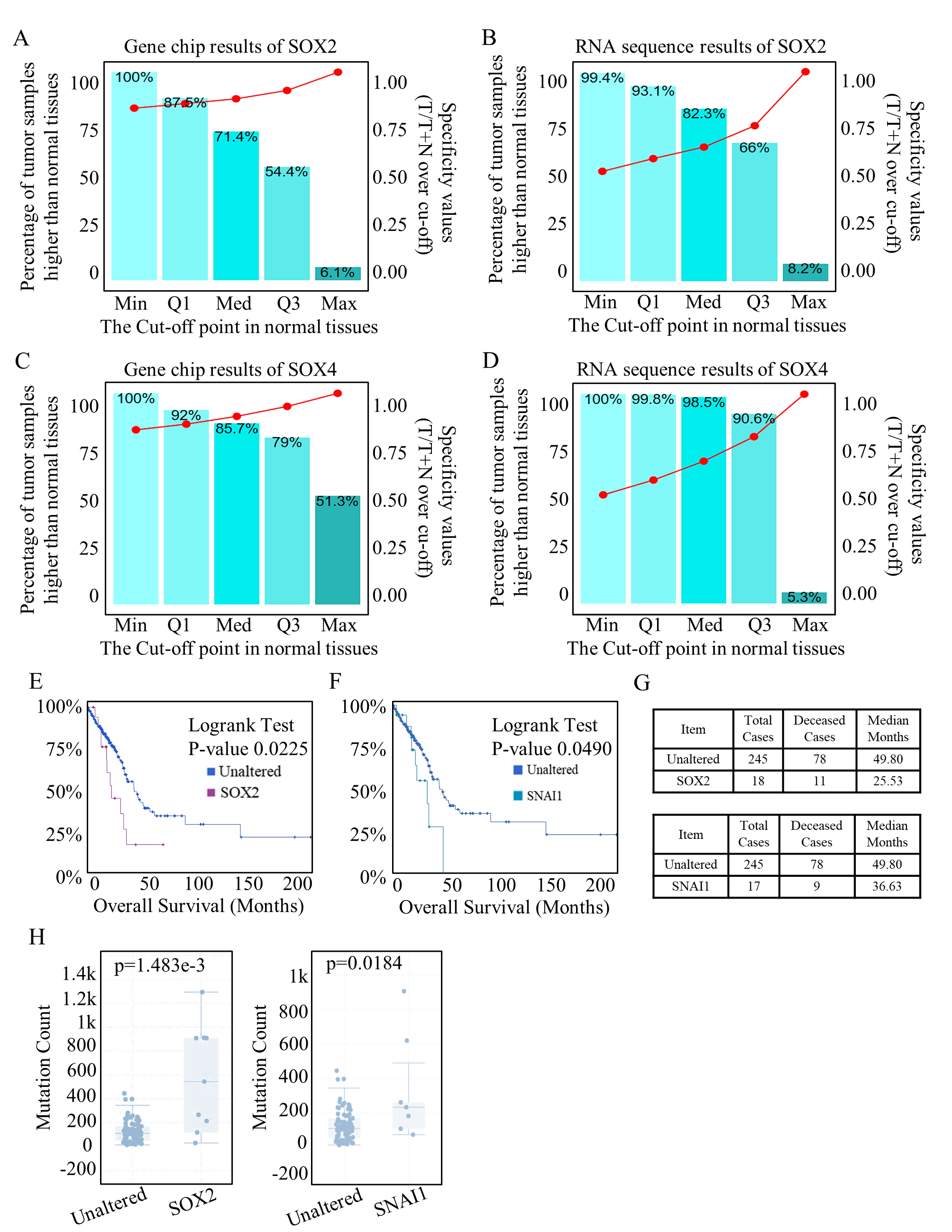

Supplement: Supplementary file 2 — Additional file 2: Supplemental Figure 2. Wnt signaling members expressing and clinical signatures. [file 12943_2023_1811_MOESM2_ESM.tif]

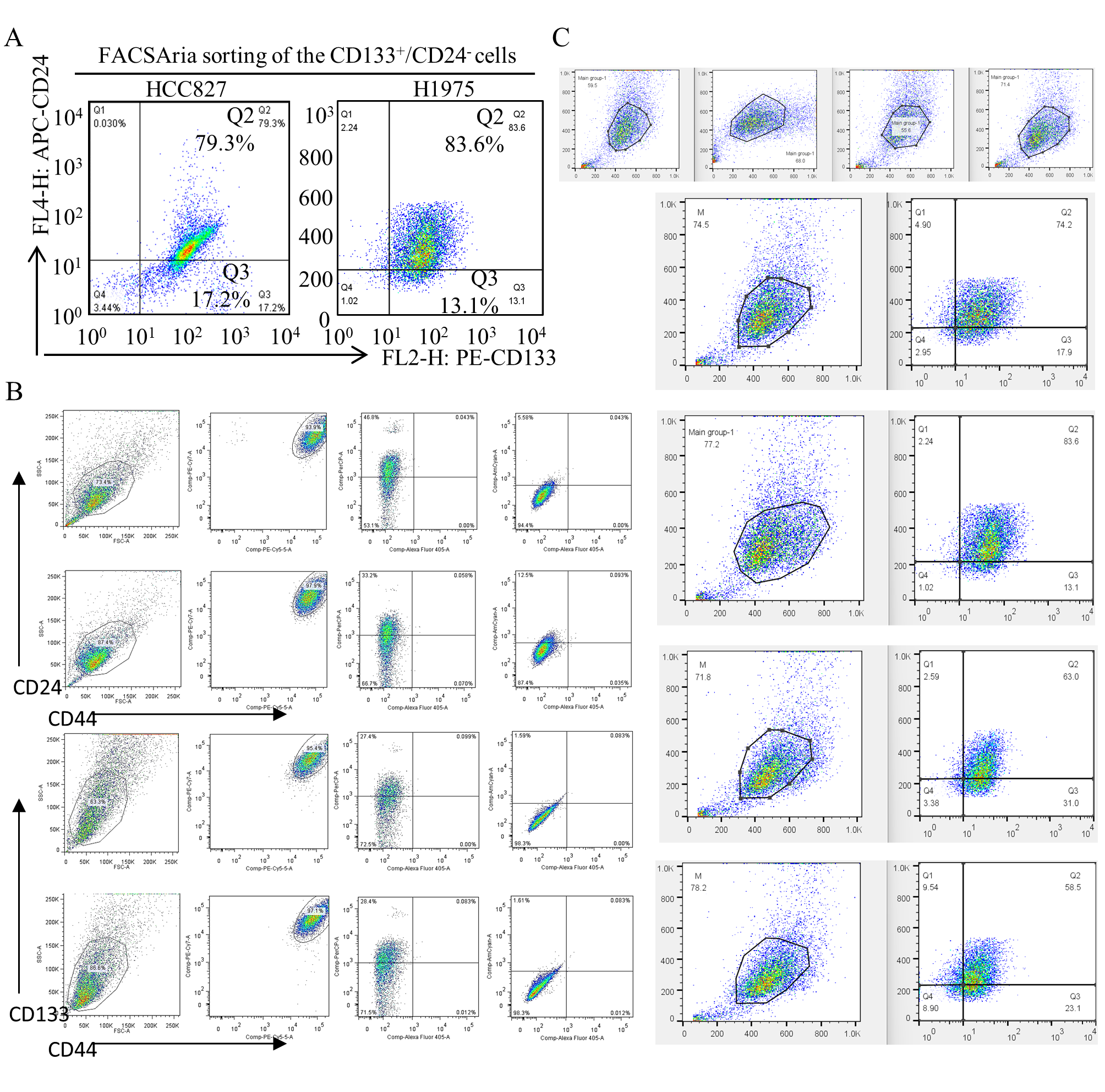

Supplement: Supplementary file 3 — Additional file 3: Supplemental Figure 3. Raw images referring to flow analysis. [file 12943_2023_1811_MOESM3_ESM.tif]

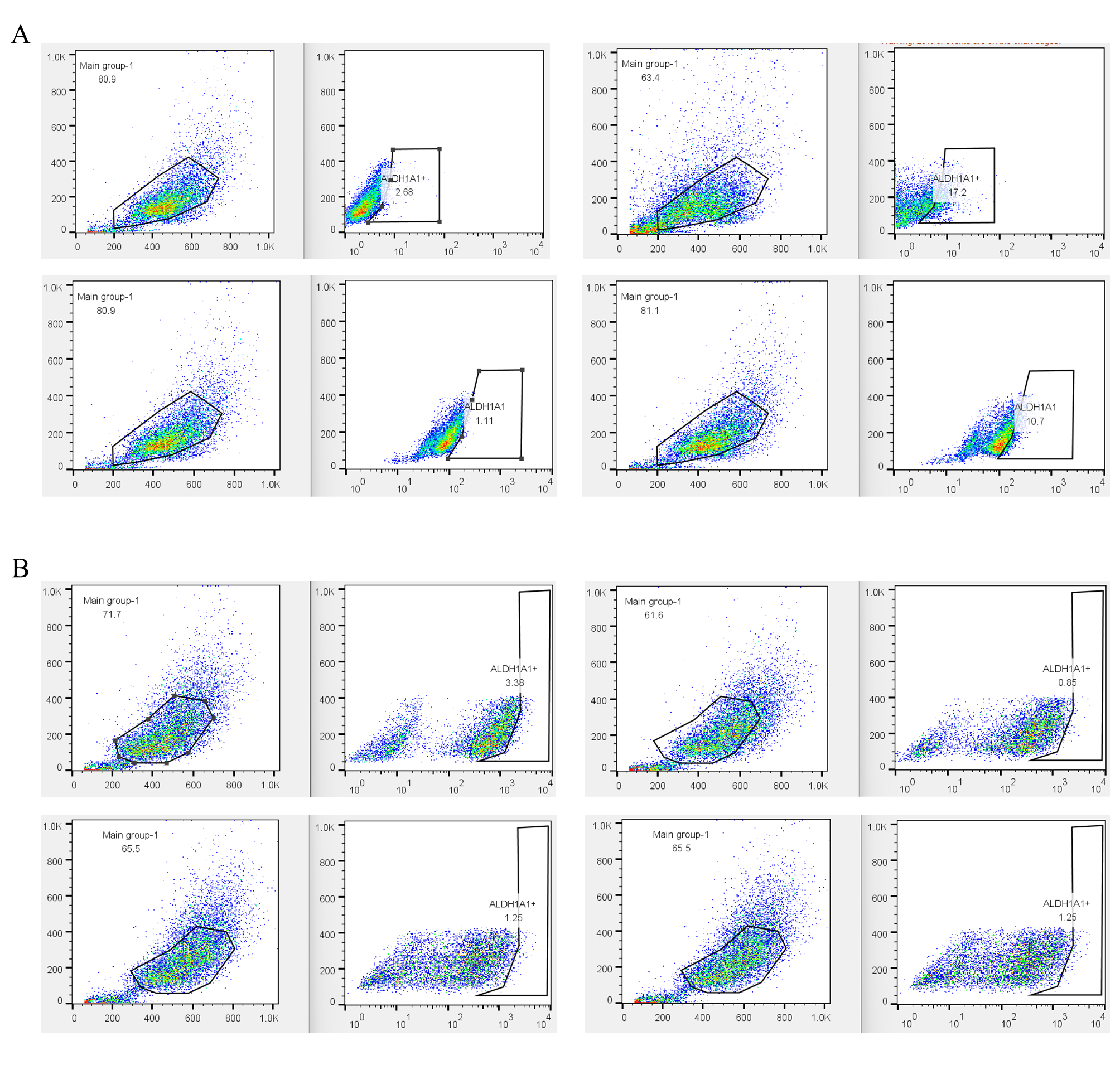

Supplement: Supplementary file 4 — Additional file 4: Supplemental Figure 4. Raw images referring to flow analysis. [file 12943_2023_1811_MOESM4_ESM.tif]

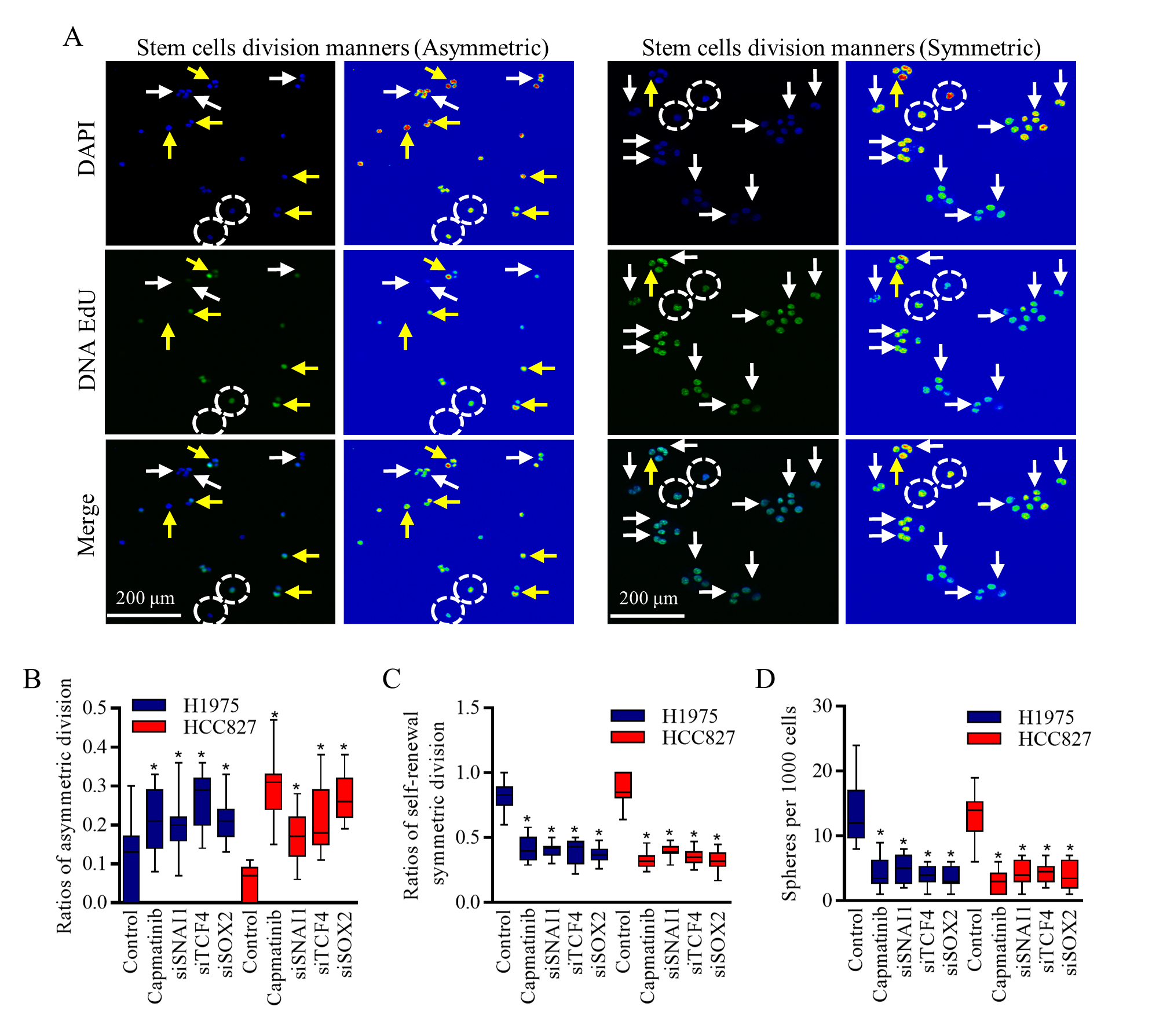

Supplement: Supplementary file 5 — Additional file 5: Supplemental Figure 5. Wnt signaling status affected the stem cells’ division. [file 12943_2023_1811_MOESM5_ESM.tif]

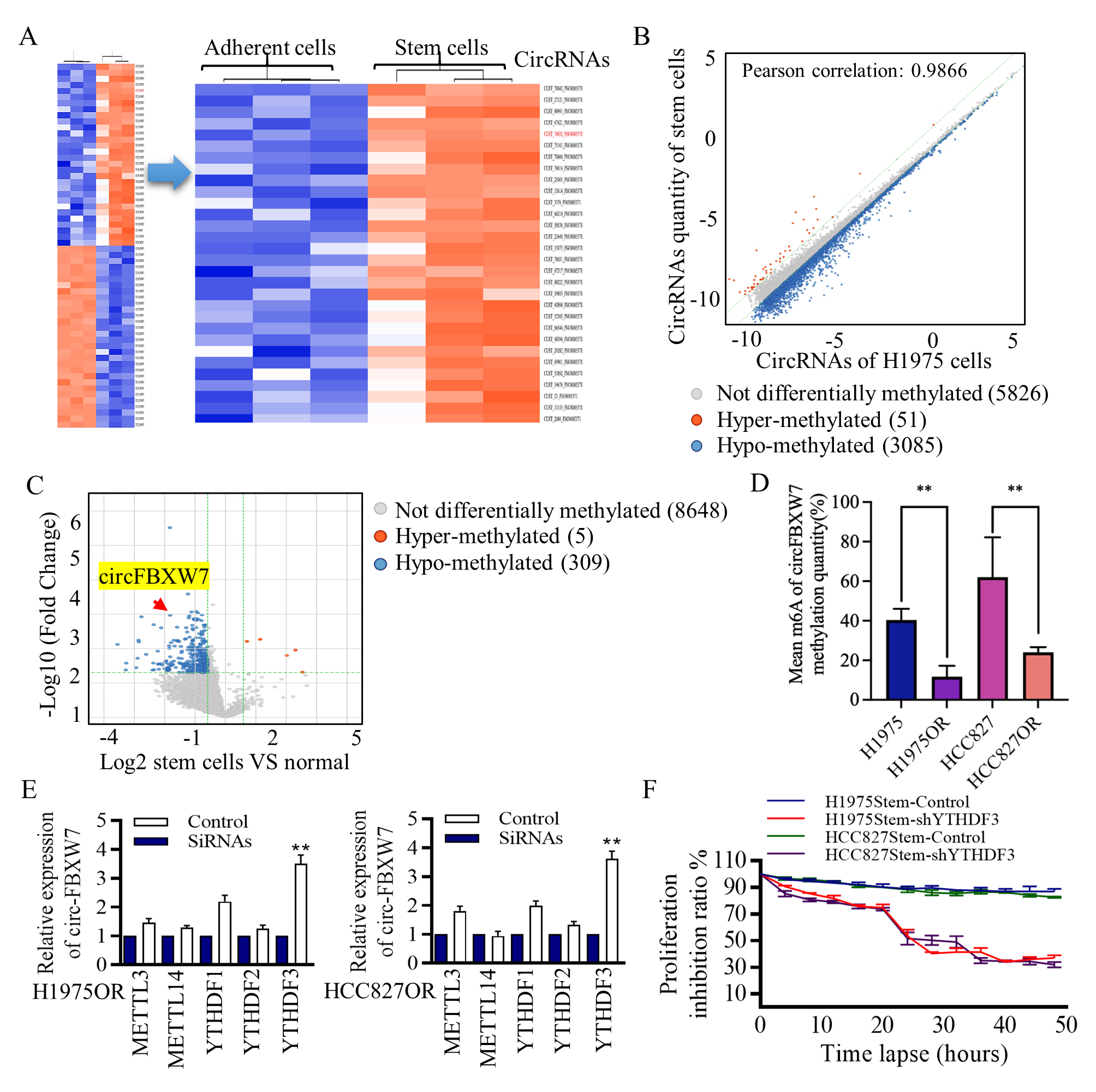

Supplement: Supplementary file 6 — Additional file 6: Supplemental Figure 6. M6A regulation of hsa-circ-0001451 (circ-FBXW7) affected the stem cells’ renewal and the consequent therapy resistance. [file 12943_2023_1811_MOESM6_ESM.tif]

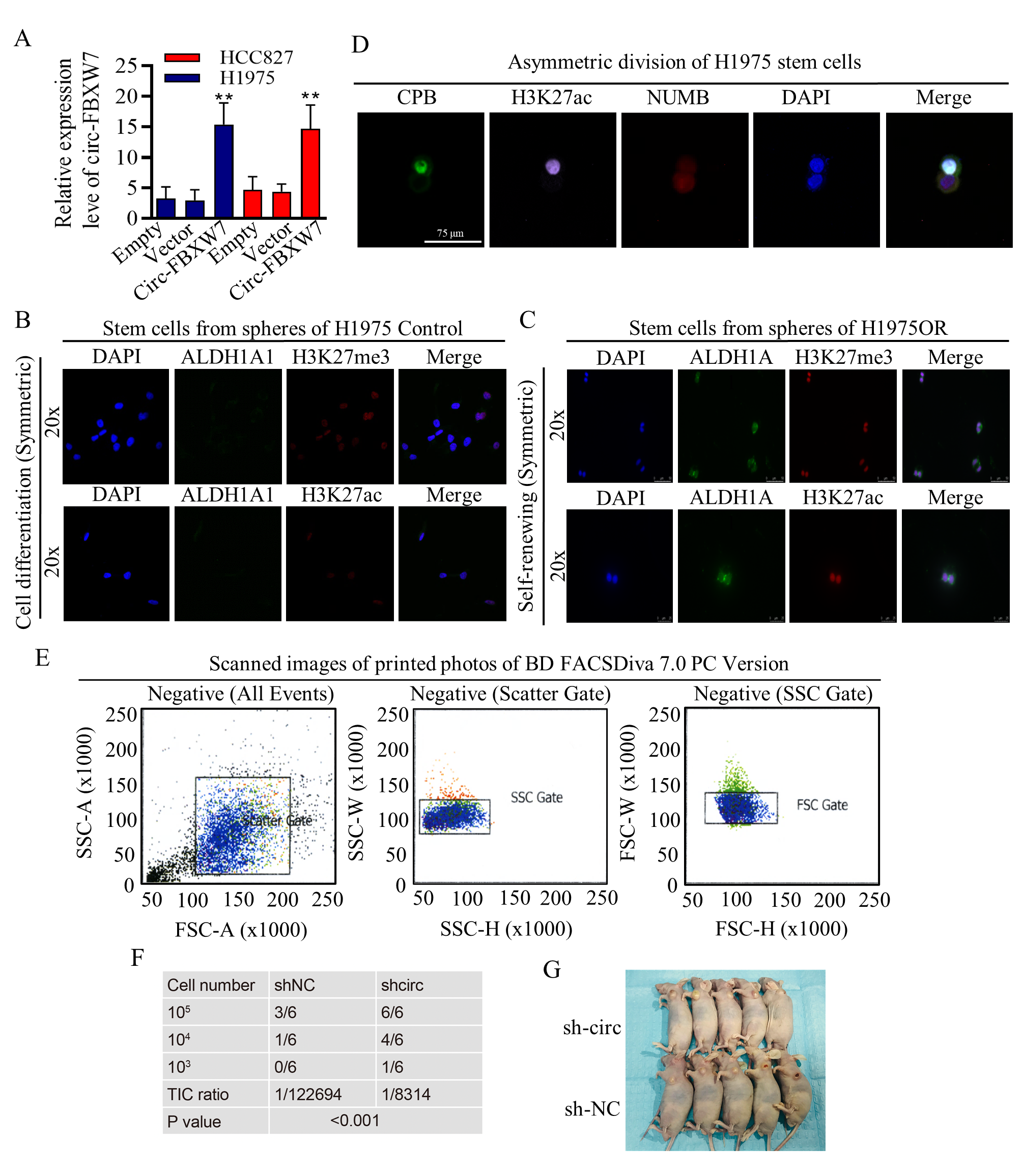

Supplement: Supplementary file 7 — Additional file 7: Supplemental Figure 7. Circ-FBXW7 controlled the cells’ division manners. [file 12943_2023_1811_MOESM7_ESM.tif]

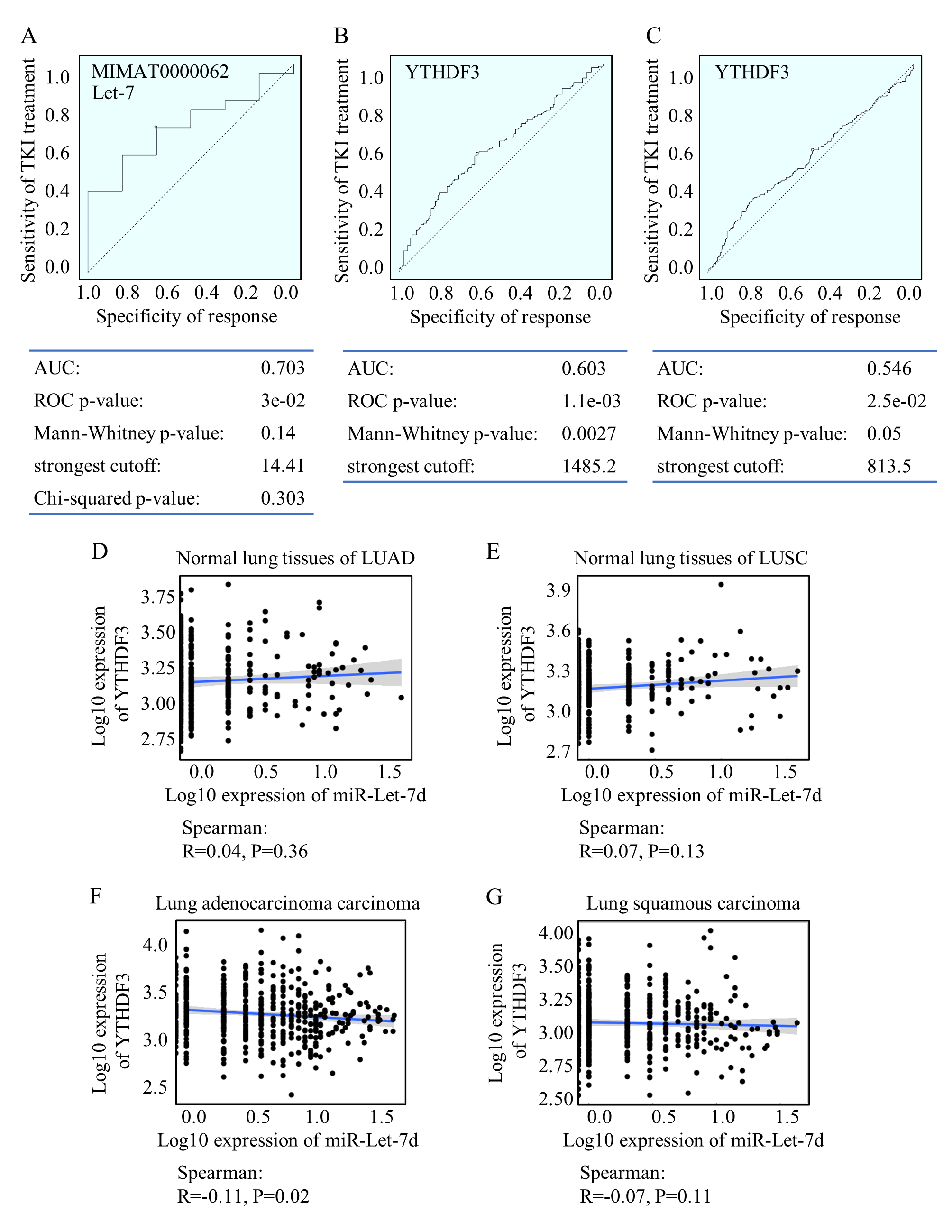

Supplement: Supplementary file 8 — Additional file 8: Supplemental Figure 8. The correlation between let-7 family of miRNAs and YTHDF3 mRNA expression. [file 12943_2023_1811_MOESM8_ESM.tif]

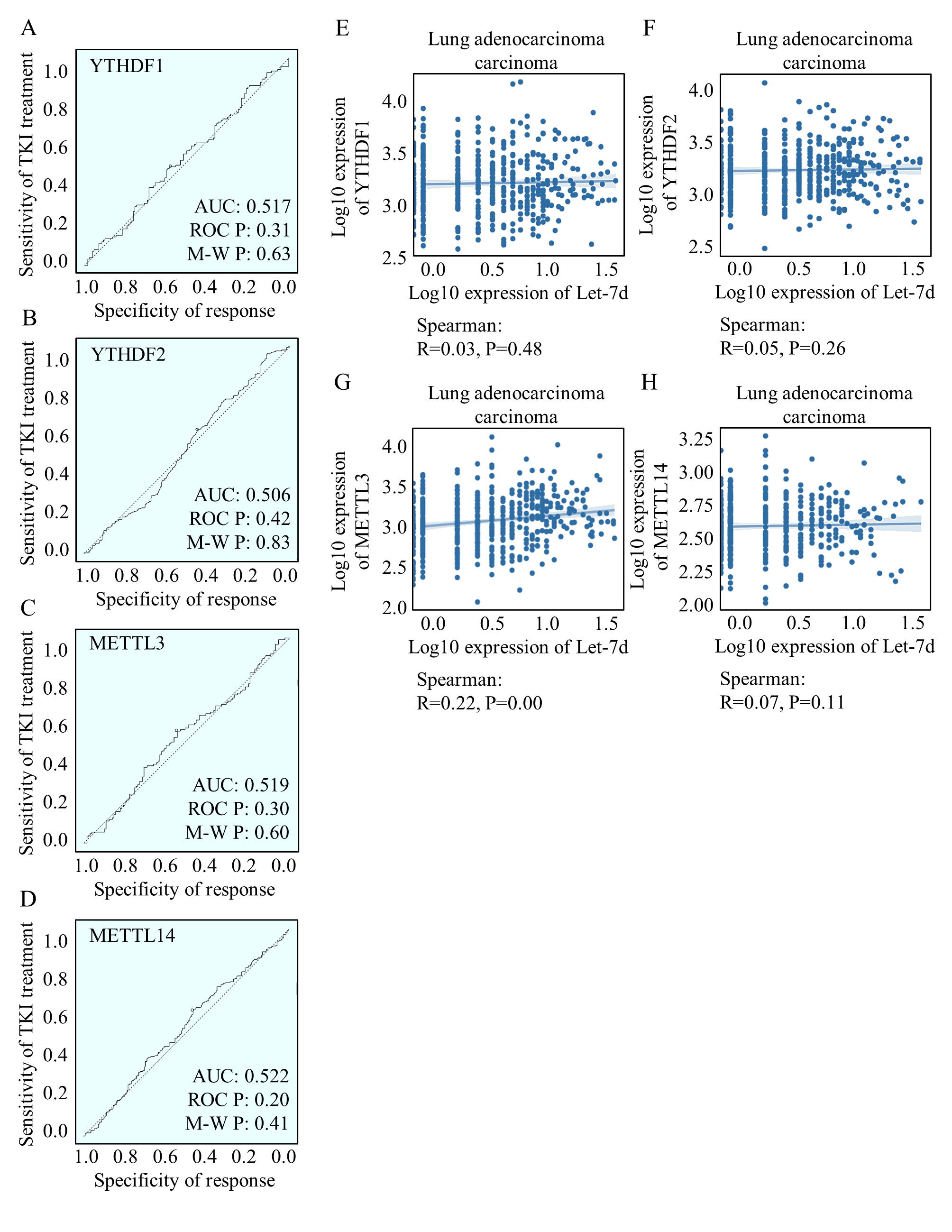

Supplement: Supplementary file 9 — Additional file 9: Supplemental Figure 9. The correlation between let-7 family of miRNAs and YTHDF3 mRNA expression. [file 12943_2023_1811_MOESM9_ESM.tif]

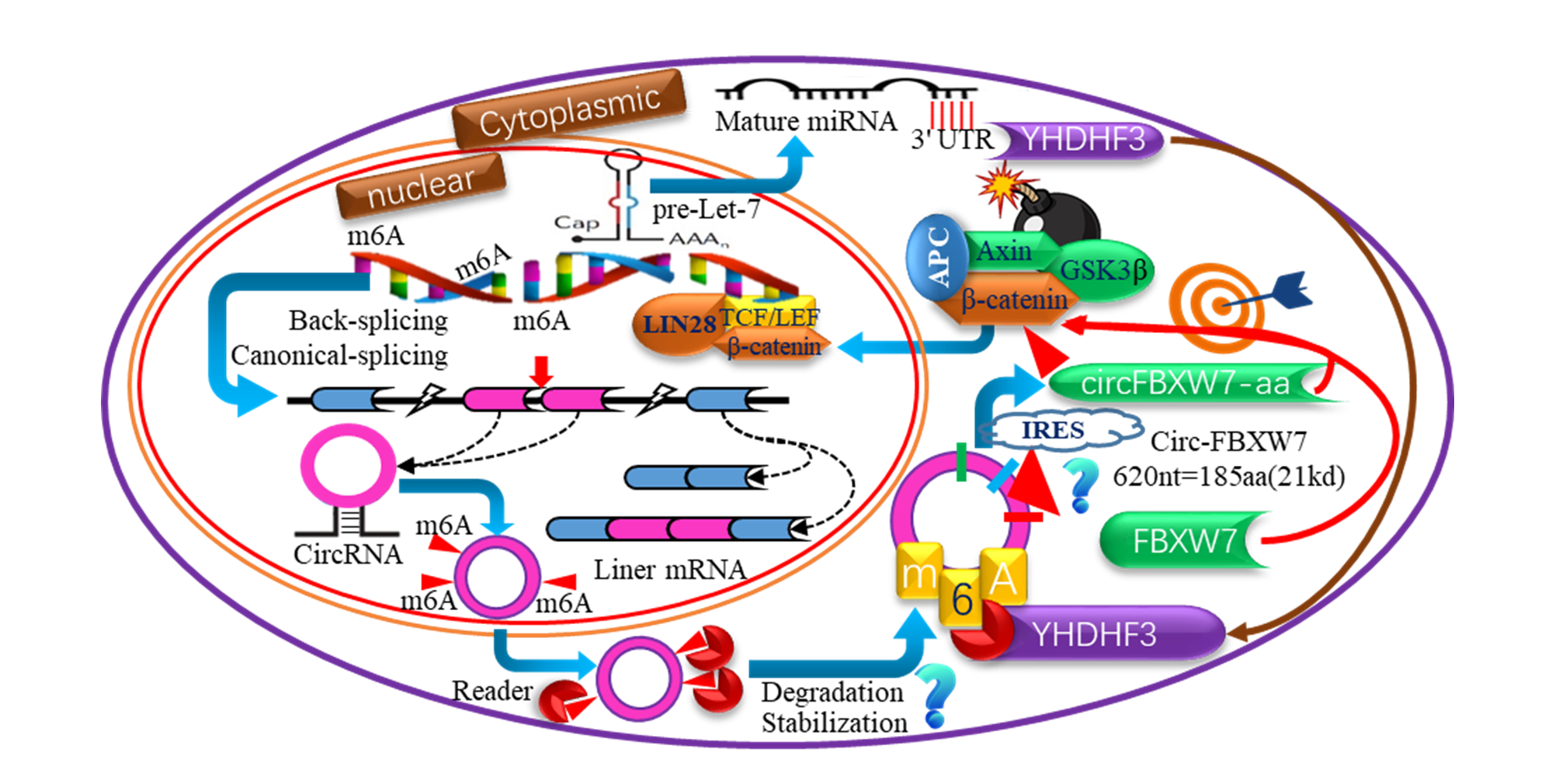

Supplement: Supplementary file 10 — Additional file 10: Supplemental Figure 10. M6A participation in circRNAs’ dysregulation and translation determined the stem cells’ therapy response [file 12943_2023_1811_MOESM10_ESM.tif]

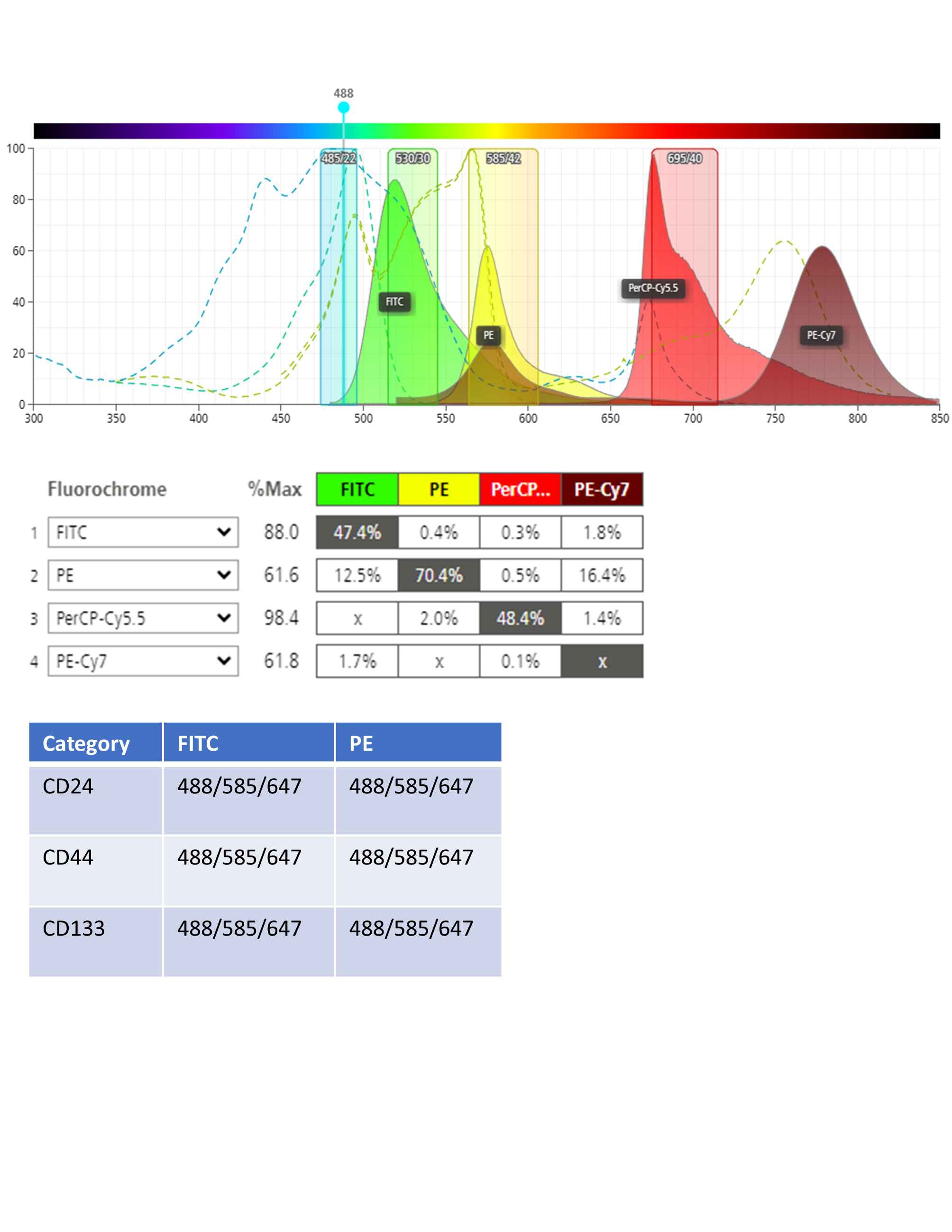

Supplement: Supplementary file 11 — Additional file 11: Supplemental Figure 11. Supporting information for flow analysis. [file 12943_2023_1811_MOESM11_ESM.tif]

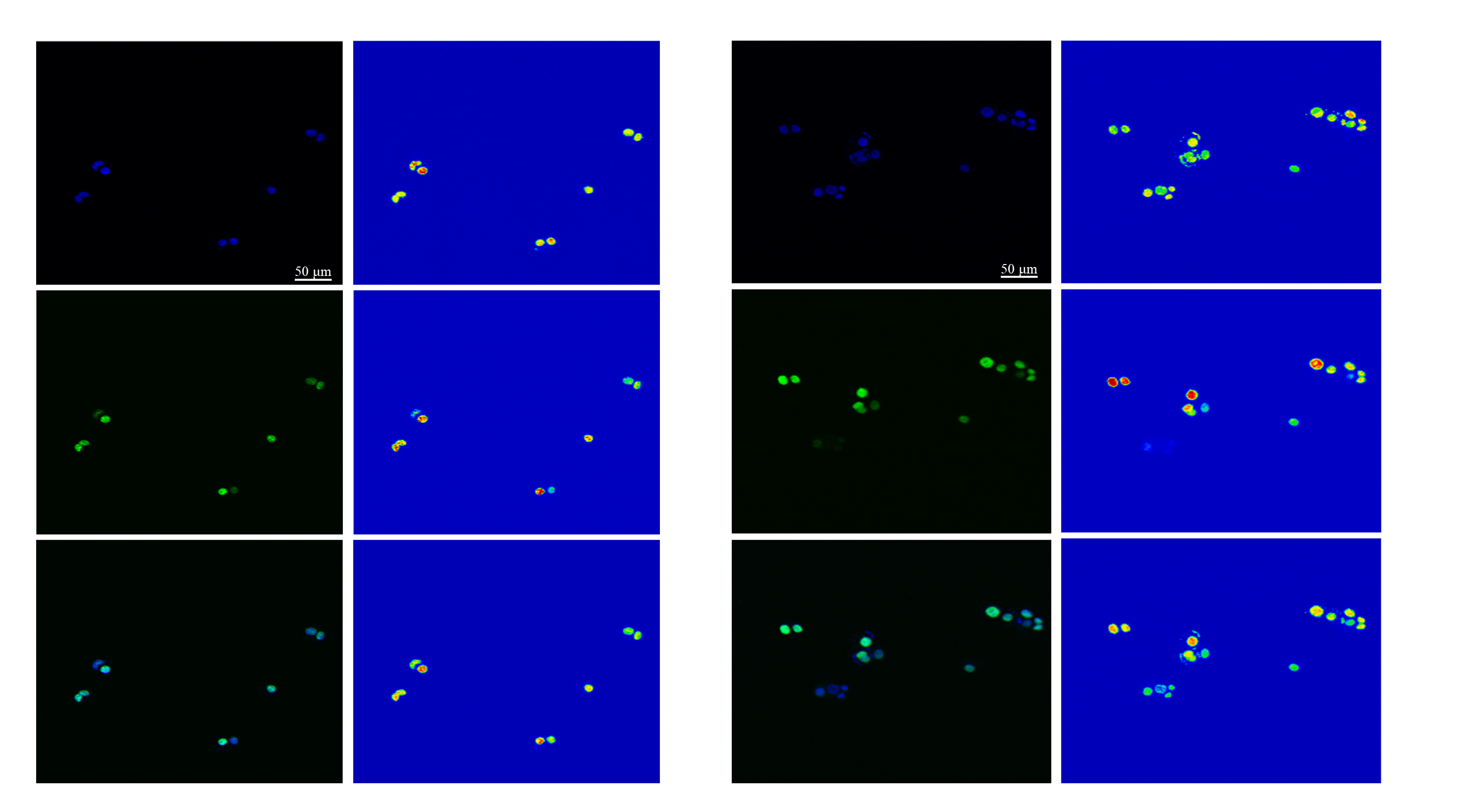

Supplement: Supplementary file 12 — Additional file 12: Supplemental Figure 12. The original images of IF experiments referring to Fig. 2. [file 12943_2023_1811_MOESM12_ESM.tif]

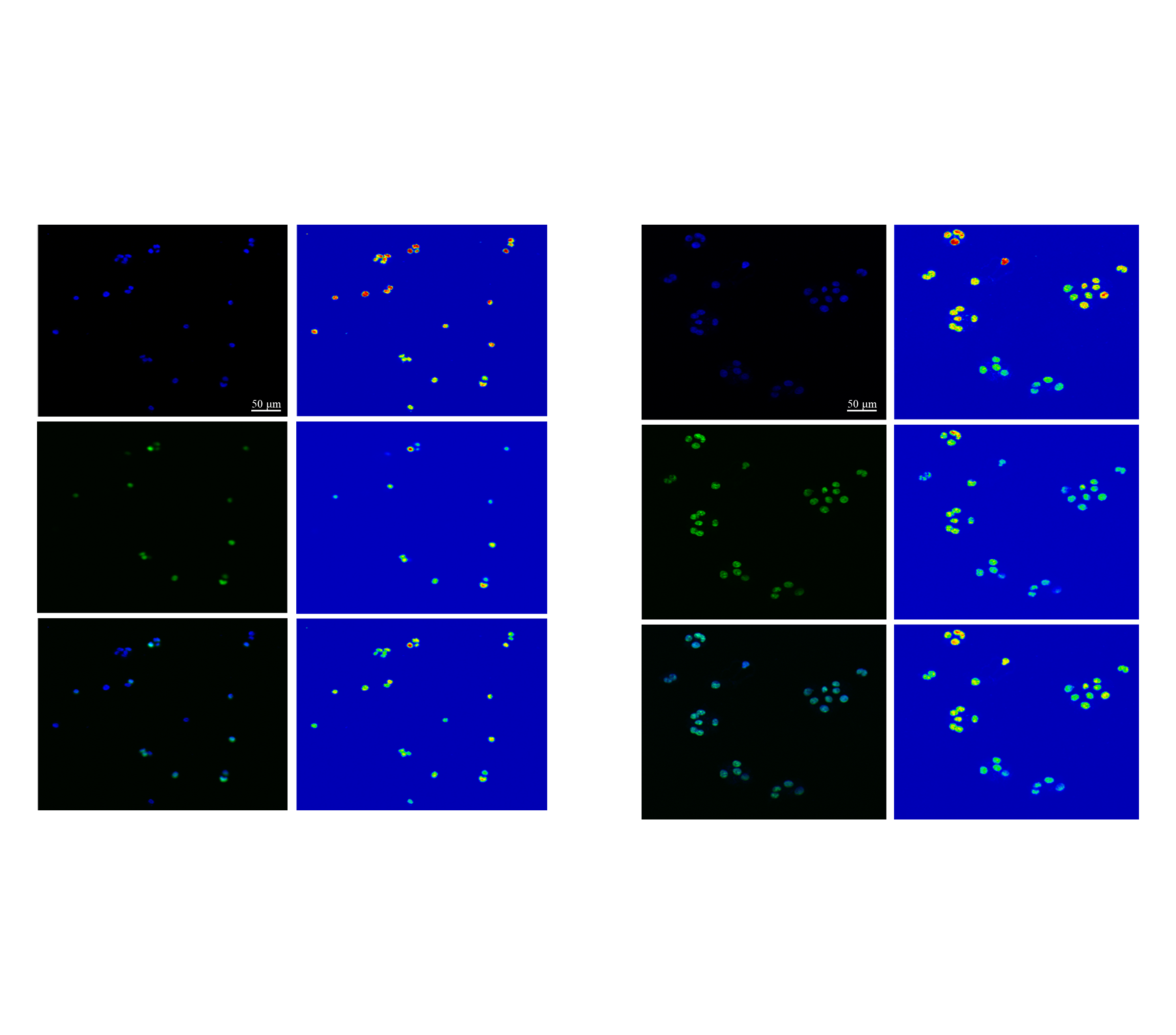

Supplement: Supplementary file 13 — Additional file 13: Supplemental Figure 13. The original images of IF experiments referring to Figure S4. [file 12943_2023_1811_MOESM13_ESM.tif]

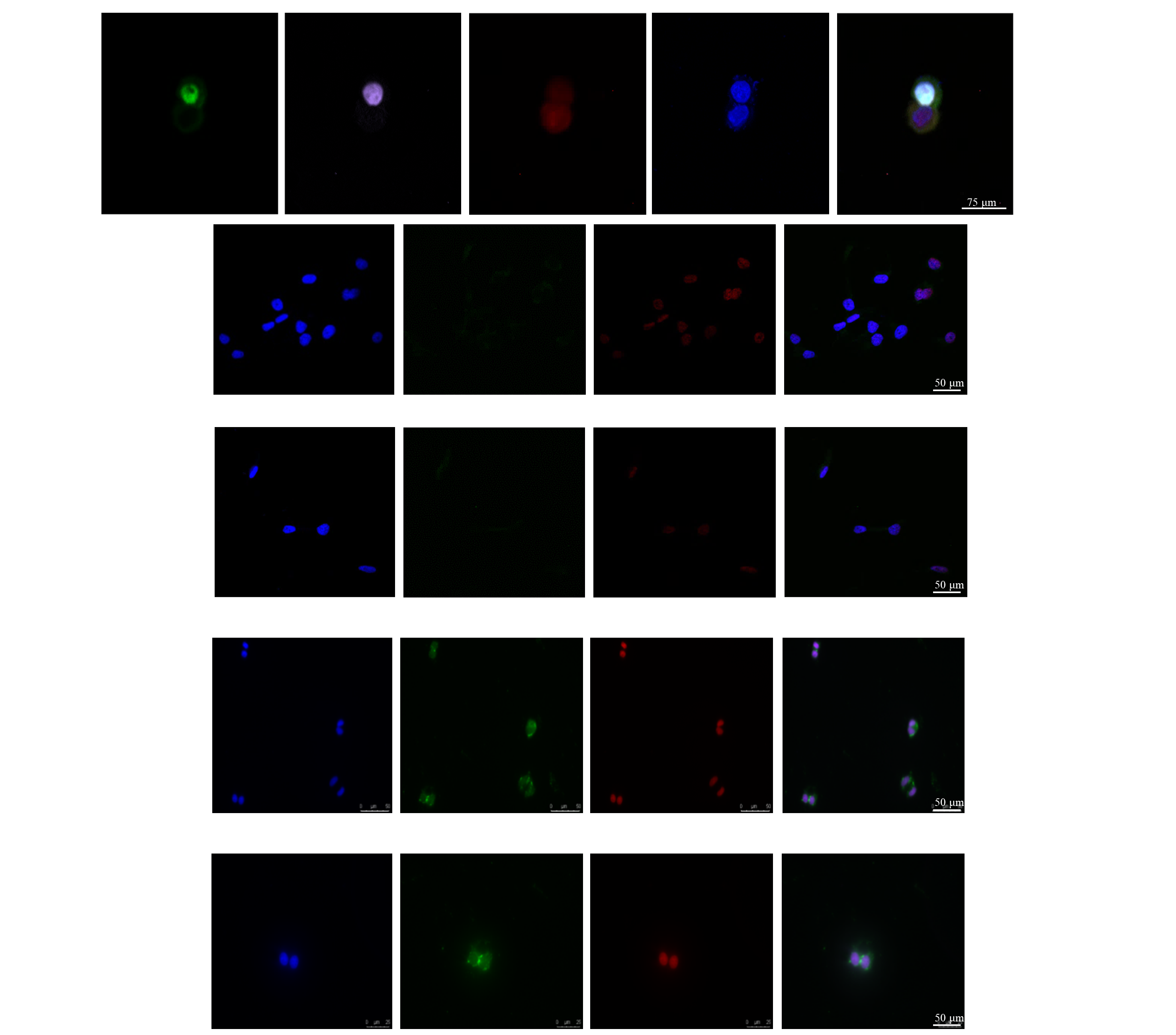

Supplement: Supplementary file 14 — Additional file 14: Supplemental Figure 14. The original images of IF experiments referring to Figure S6. [file 12943_2023_1811_MOESM14_ESM.tif]
